# Preclinical Anxiolytic- and Antidepressant-like Effects of α,α-Trehalose, a Bioactive Compound Identified in *Psilocybe cubensis*

**DOI:** 10.3390/molecules31193383

**Published:** 2026-09-23

**Authors:** Aylín Rocío Tabal-Robles, María Eva González-Trujano, Gabriel Fernando Moreno-Pérez, Alberto Hernandez-Leon, Susana Rojas-Lima, David Martínez-Vargas, Hiram Luna-Munguía, Raúl Iván Escamilla-Orozco, Laura Guzmán-Dávalos, José G. Alvarado-Rodríguez, J. Martín Torres-Valencia

**Affiliations:** 1 Laboratorio de Neurofarmacología de Productos Naturales, Dirección de Investigaciones Biomédicas en Salud Mental, Instituto Nacional de Psiquiatría Ramón de la Fuente Muñiz, Calz. México-Xochimilco 101, Col. San Lorenzo Huipulco, Tlalpan, Ciudad de México 14370, Mexico; ta260266@uaeh.edu.mx (A.R.T.-R.); mmspoodles@gmail.com (G.F.M.-P.); albertoh-leon@hotmail.com (A.H.-L.); 2 Área Académica de Química, Universidad Autónoma del Estado de Hidalgo, Km. 4.5 Carretera Pachuca-Tulancingo, Mineral de la Reforma 42184, Mexicojgar@uaeh.edu.mx (J.G.A.-R.); 3 Laboratorio de Neurofisiología del Control y la Regulación, Dirección de Investigaciones Biomédicas en Salud Mental, Instituto Nacional de Psiquiatría Ramón de la Fuente Muñiz, Calz. México-Xochimilco 101, Col. San Lorenzo Huipulco, Tlalpan, Ciudad de México 14370, Mexico; davmv2@gmail.com; 4 Departamento de Neurobiología Conductual y Cognitiva, Instituto de Neurobiología, Universidad Nacional Autónoma de México, Campus UNAM-Juriquilla, Querétaro 76230, Mexico; hiram_luna@yahoo.com; 5 Servicios Clínicos, Dirección de Servicios Clínicos, Instituto Nacional de Psiquiatría Ramón de la Fuente Muñiz, Calz. México-Xochimilco 101, Col. San Lorenzo Huipulco, Tlalpan, Ciudad de México 14370, Mexico; rescam01@gmail.com; 6 Departamento de Botánica y Zoología, Universidad de Guadalajara, Zapopan 45200, Mexico; laura.guzman@academicos.udg.mx

**Keywords:** α,α-trehalose, anxiety, depression, electrocorticogram, *Psilocybe cubensis*

## Abstract

The genus *Psilocybe* has been characterized by central nervous system (CNS) effects and as a source of therapeutic drugs such as the alkaloids psilocybin and psilocin—secondary metabolites that produce rapid anxiolytic and antidepressant effects in preclinical and clinical studies. Furthermore, these mushrooms also contain bioactive primary metabolites, for example, the interesting case of α,α-trehalose. To explore the effects of this disaccharide, it was isolated and characterized using phytochemical and spectroscopic analyses. Subsequently, its anxiolytic- and antidepressant-like properties were assessed and corroborated by in vivo behavioral studies using experimental models for anxiety (open-field, hole-board, and plus-maze tests) and depression (forced swimming test complemented by a preliminary rota-rod test). Electrocorticographic in situ recordings and nuclear magnetic resonance analysis were also explored. Finally, an in silico docking analysis was performed to determine the involvement of the 5-HT_1A_ serotonin receptor as one of the main targets identified in the CNS activity of *Psilocybe* species. The evidence shows that α,α-trehalose is one bioactive metabolite that promotes significant anxiolytic- and antidepressant-like activities mediated by early brain changes and involving an indirect participation of 5-HT_1A_ receptors. In conclusion, α,α-trehalose represents a potential natural drug for mental health, such as in the treatment of anxiety, depression, or their comorbidity.

## 1. Introduction

Major Depressive Disorder (also known as depression) is a common mental disorder, either alone or in comorbidity with anxiety, that involves a depressed mood or loss of pleasure or interest in activities for extended periods of time; this affects all aspects of a person’s life, including participation in the community, at home, at work, and at school. Anxiety is characterized by excessive worry, fear, and restlessness, while depression is associated with persistent sadness, anhedonia, and loss of energy. A depressive or anxiety episode can be categorized as mild, moderate, or severe depending on the number and severity of symptoms, as well as the impact on the individual’s functioning [1]. It is estimated that by the year 2030, depression will become the leading cause of disability [2]. Both are multifactorial disorders that constitute a priority challenge for contemporary public health, encompassing biological, psychological, and social components; because of this, efficacy and tolerability might vary among individuals relative to the general population [3].

These common mental disorders share neurobiological mechanisms related to alterations in neurotransmitters such as serotonin, norepinephrine, dopamine, and gamma-aminobutyric acid (GABA), as well as the dysregulation of the hypothalamic–pituitary–adrenal (HPA) axis and changes in brain regions such as the amygdala, hippocampus, and prefrontal cortex, which are involved in emotional regulation and decision-making [4,5]. First-line treatments include selective serotonin reuptake inhibitors (SSRIs) and serotonin, and norepinephrine reuptake inhibitors (SNRIs), while benzodiazepines can be employed for anxiety management due to their action on GABA_A_ receptors [6]. Although there are anxiolytics and/or antidepressant drugs with similar or different mechanisms of action, they are not always effective and, conversely, can cause various adverse effects, as is the case with SSRIs, SNRIs, and tricyclic antidepressants (TCAs) such as imipramine (IMI) [3]. Likewise, cognitive behavioral therapy and other psychological interventions have demonstrated significant efficacy in both disorders, especially when combined with strategies such as physical activity and mindfulness [7]. SSRIs have the best benefit–risk profile, making them suitable as first-line treatments, while tricyclics are highly effective but less tolerated than SSRIs and placebo [3]. Clinical studies have reported that IMI produces a significant advantage in measures of depressive symptoms, global disease severity, and self-reported social and occupational functioning [8].

Natural alternatives are often considered by individuals suffering from this condition, such as *Psilocybe* species, which represent one of the oldest sources of psychotropics documented in history through cave paintings dating back more than 7000 years and associated with psychopharmacological properties [9]. Fray Bernardino de Sahagún was one of the first to write about psychotropic mushrooms in Mexico and America by describing the customs of the Aztecs, referring to mushrooms as “teonanácatl” (teo: sacred; nanácatl: mushroom) [10]. Ancient historical records of the therapeutic properties of *Psilocybe* mushrooms can be found in the Florentine Codex, written in the 16th century in Nahuatl and Spanish. Gordon Wasson and his wife Valentina Pavlovna (researchers of mycophilic communities) arrived in Huautla de Jiménez, Oaxaca, and learned about the rituals in which the 61-year-old healer María Sabina (an expert in rituals and healing with mushrooms) participated [11]. Indigenous medicine conceives the therapeutic effects of whole *Psilocybe* mushrooms as acting in “synergy” with other components, i.e., through the entourage effect.

The genus *Psilocybe* is widely distributed throughout the world, and its species count fluctuates between 277 and 300 [12,13]. Chemical analysis of this genus has reported alkaloids as the main nature of its bioactive compounds. A substantial body of scientific evidence regarding its potential participation in the biological activity of *Psilocybe* mushrooms points to psilocybin and psilocin, which were first identified by Albert Hofmann. After 63 years of chemical studies on *Psilocybe* species, it seemed that the alkaloids present in them possessed only a tryptamine structure. However, the presence of some beta-carboline producers has also been mentioned as part of the relevant constituents in *Psilocybe* species [14], such as harmine and harmane (in lower quantities), found in the carpophores of *Psilocybe cyanescens*, *Psilocybe semilanceata*, and two *Psilocybe cubensis* isolates, as well as in *Psilocybe mexicana* (both sclerotia and mycelium) and in *Psilocybe cubensis* mycelium [15]. Although chemical studies on these types of mushrooms have focused on the search for hallucinogenic bioactive constituents, which has led to the knowledge of psilocybin and psilocin as the principal ones [2], information on the chemical composition and biological activity of metabolites other than these commonly and well-described indole tryptamine alkaloids is missing in the literature. This is the case with the disaccharide α,α-trehalose, which has been reported due to its applications in the food, cosmetic, and medical industries [16]. The originality of this study lies in the results regarding the participation of a primary metabolite from the *Psilocybe cubensis* mushroom. While this metabolite can be found in other fungi, insects, or plants, the evidence that its presence as an isolated product improves behavioral and brain responses in a preclinical model of two disorders commonly associated with impaired mental health, such as anxiety and depression, is a valuable contribution to what has been reported for its therapeutic use. Since the effect of *Psilocybe cubensis* has been emphatically linked to the presence of indole alkaloids, the integration of other bioactive components into the knowledge base remained to be explored in order to understand their relevance and benefits for mental health.

Because α,α-trehalose is one of the primary metabolites in the bioactive polar extracts of *Psilocybe cubensis* [17] and is also recognized as a bioactive compound that produces effects on the central nervous system (CNS) to combat neurodegenerative disorders, the aim of this research was to explore its anxiolytic and antidepressant-like properties using in situ electrocorticographic (ECoG) recordings and Nuclear Magnetic Resonance (NMR) analysis, as well as behavioral assessment in in vivo murine models, following its isolation and characterization through phytochemical studies. Additionally, to explore the participation of the 5-HT_1A_ receptor—as an inhibitory receptor involved in anxiety and depression disorders—using molecular docking and in vivo analysis.

## 2. Results

### 2.1. Phytochemical Analysis

#### Isolation and Characterization of α,α-Trehalose

A biphasic partitioning of the methanol (MeOH) extract of *Psilocybe cubensis* (Earle) Singer led to the organic fraction (OF, obtained with hexane) and aqueous fraction (AQ) [18]. Separation by column chromatography (CC) of the AQ phase using microcrystalline cellulose and 1-butanol saturated with H_2_O led to the isolation of α,α-trehalose (Figure 1), a known symmetric disaccharide composed of two D-glucose units (Figure 2). This compound was characterized by its physical and spectroscopic properties and by comparison with reported data [19]. Additionally, its ^1^H and ^13^C NMR spectra were directly compared with those of a commercial authentic sample of α,α-trehalose (Sigma-Aldrich) (see Supplementary Materials).

Due to its symmetry (two α-D-glucopyranose units linked), in the NMR spectra, each signal was assigned to integrate as twice as many protons and carbons. Finally, an X-ray diffraction study was performed on the obtained crystal of this disaccharide, confirming its structure (Figure 2). Its crystallographic data were consistent with those previously reported in the literature [20]. The percentage of α,α-trehalose in the MeOH extract was determined by absolute quantitative ^1^H NMR spectroscopy, a reliable method for characterizing the compound’s purity. The concentration of α,α-trehalose in this extract was 14% (see Supplementary Materials). Therefore, 1 g of the MeOH extract contains 140 mg of α,α-trehalose, while 1 g of the dried mushroom contains 24.36 mg of this metabolite.

### 2.2. Pharmacological Study

#### 2.2.1. Anxiolytic-like Effects of α,α-Trehalose and Participation of 5-HT_1A_ Serotonin Receptor

In the open-field test, no differences were observed in the ambulatory activity of mice that received treatment with the reference drug buspirone (4 mg/kg, i.p.) nor with α,α-trehalose at all doses tested (1, 10, and 100 mg/kg, i.p.) (Figure 3A). In contrast, a significant but dose-independent reduction was observed in the escape-like behavior (rearing performance) at doses of 10 and 100 mg/kg, i.p., of α,α-trehalose administered, without changes in the reference drug buspirone (F_6,35_ = 4.30, *p* = 0.0024) (Figure 3B). Similarly to the rearing behavior, α,α-trehalose produced a significant dose-independent reduction in the behavioral response in the hole-board test that resembled the significant effect of buspirone (F_6,35_ = 5.34, *p* = 0.0005) (Figure 3C). No changes were observed following the administration of WAY-100635 (WAY, 1 mg/kg, i.p.) alone—α 5-HT_1A_ serotonin receptor antagonist known to interact with the tryptamine alkaloids of *Psilocybe cubensis*, psilocybin and psilocin—in any of the three tests (Figure 3A).

To reinforce the anxiolytic-like activity of α,α-trehalose, the elevated plus-maze test was performed. All parameters evaluated showed a significant and dose-independent increase in the presence of α,α-trehalose (Figure 4A–D) and in an equivalent manner to those observed with buspirone (4 mg/kg, i.p.), such as in latency (F_6,35_ = 2.84, *p* = 0.02) (Figure 4A) and in the average time in the open arms (F_6,35_ = 3.58, *p* = 0.007) (Figure 4C). The effects of α,α-trehalose were greater than the reference drug in the time spent in the open arms (F_6,35_ = 8.20, *p* < 0.0001) (Figure 4B) and also in the number of entries into the open arms (F_6,35_ = 4.71, *p* = 0.0013) (Figure 4D).

Regarding the involvement of the 5-HT_1A_ serotonin receptor, the antagonist did not modify the response observed in the vehicle group, except in the parameter of latency (Figure 4A). In contrast, the presence of α,α-trehalose (100 mg/kg, i.p.) significantly increased the response in all parameters, which generally remained significant even in the presence of the serotonergic antagonist, mainly in the number of entries (Figure 4D) and in the average time (Figure 4C) and time spent by the mice (Figure 4B) in the open arms.

#### 2.2.2. Antidepressant-like Effects of α,α-Trehalose

In the rota-rod test, no changes in motor coordination were observed across any treatment tested (Figure 5A). In the Forced Swimming Test (FST), α,α-trehalose significantly reduced immobility time in a dose-independent manner at doses of 10 and 100 mg/kg, i.p. (F_6,35_ = 7.05, *p* < 0.0001) (Figure 5C) by increasing swimming behavior (F_6,35_ = 7.04, *p* < 0.0001) (Figure 5C), mimicking the effect of the reference drug Imipramine (IMI, 10 mg/kg, i.p.) (Figure 5C and Figure 5B, respectively). Climbing behavior was significantly increased only in the presence of IMI (10 mg/kg, i.p.) (F_6,35_ = 8.07, *p* < 0.0001) (Figure 5D).

The administration of WAY (1 mg/kg, i.p.) alone did not produce changes in the FST behavioral response, except in the climbing behavior, where, both alone and in the combination with α,α-trehalose (100 mg/kg, i.p.), it produced a significant increase in this response (Figure 5D). Similar to the anxiety tests, the presence of WAY partially attenuated the response observed in the presence of α,α-trehalose alone (Figure 5B,C).

#### 2.2.3. ECoG Analysis of α,α-Trehalose and the Involvement of the 5-HT_1A_ Serotonin Receptor

Power spectral analysis of the left frontal cortex revealed frequency-band-dependent differences across treatments (Figure 6). Notably, α,α-trehalose treatment induced an increase in low-frequency power, particularly within the 1–4 Hz (delta) and 10–15 Hz (sigma) frequency bands. This effect was most pronounced in the combined WAY + α, α-trehalose group, which exhibited a marked enhancement of slow-wave activity and a more sustained pattern of cortical synchronization compared to the other treatments. In contrast, the vehicle group maintained a relatively stable spectral distribution, whereas buspirone or WAY alone induced more subtle changes.

In the delta band, a significant effect was observed (F_4.000,11.76_ = 22.66, *p* < 0.0001), with an increase in absolute power across all treated groups: buspirone (4 mg/kg, i.p., *p* < 0.001), WAY (1 mg/kg, i.p., *p* < 0.05), α,α-trehalose (100 mg/kg, i.p., *p* < 0.001), and the combination of WAY (1 mg/kg, i.p.) + α,α-trehalose (100 mg/kg, i.p.) (*p* < 0.0001), compared with the vehicle group. Notably, the combined treatment of WAY + α,α-trehalose produced a significantly greater increase than α,α-trehalose alone (*p* < 0.05) (Figure 7A). To assess deviations from additivity between groups WAY and α,α-trehalose, the observed experimental effect of co-administration was compared with the theoretical sum of the individual treatment effects. This analysis revealed that the combined treatment produced an effect that was significantly different from the theoretical sum (t_10_ = 2.54, *p* = 0.0294), indicating a supra-additive interaction (Figure 7A).

In the theta band, significant differences were also observed (F_4.000,12.18_ = 9.21, *p* = 0.0012). Individual treatments did not produce a significant effect; however, the combined treatment (WAY + α,α-trehalose) induced a significant increase compared with the vehicle (*p* < 0.001), α,α-trehalose alone (*p* < 0.05), and the theoretical sum (t_10_= 3.909, *p* = 0.0029) (Figure 7B).

Treatments also reached significance in the sigma band (F_4,12.19_ = 19.39, *p* < 0.0001). Both WAY (1 mg/kg, i.p.) and α,α-trehalose (100 mg/kg, i.p.) increased absolute power compared with the vehicle (*p* < 0.01 for both). The combined treatment elicited a robust potentiation of this effect, which was significantly greater than α,α-trehalose alone (*p* < 0.05) and differed significantly from the theoretical sum (t_10_ = 4.036, *p* = 0.0024) (Figure 7C).

A moderate effect was observed in the beta band (F_4,12.29_ = 10.93, *p* = 0.0005), where only the combination of WAY (1 mg/kg, i.p.) +α,α-trehalose (100 mg/kg, i.p.) induced a significant increase compared with vehicle or α,α-trehalose alone (*p* < 0.05 for both), which also differed significantly from the theoretical sum (t_10_ = 3.028, *p* = 0.0127) (Figure 7D).

Conversely, in the gamma band, a significant power reduction was observed in animals treated with buspirone (4 mg/kg, i.p.) or α,α-trehalose (100 mg/kg, i.p.) compared with the vehicle (*p* < 0.05 for both; F_4,25_ = 4.665, *p* = 0.0060) (Figure 7E). These findings indicate that α,α-trehalose promotes cortical synchronization characterized by enhanced low-frequency oscillations in the left frontal cortex, an effect that is potentiated by 5-HT_1A_ receptor antagonism.

It is worth noting that no signs of neurotoxicity or abnormal electrophysiological patterns were observed during the recordings in any treatment group.

#### 2.2.4. Molecular Docking Analysis of α,α-Trehalose and Serotonin (5-HT) with the 5-HT_1A_ Serotonin Receptor

First, 5-HT was predicted to bind within the canonical orthosteric pocket of the 5-HT_1A_ receptor with a Vina affinity score = −6.1 kcal/mol. The selected interaction map highlighted conventional hydrogen bonds with Thr121 and Tyr390; a predicted salt bridge with Asp116; a π–π T-shaped interaction with Phe361; and π–alkyl contacts involving Val117, Ile189, Ala203, and Cys120. Additional surrounding residues shown in the interaction map included Phe362, Ser199, Ala365, Ile124, and Asn386 (Figure 8). This orthosteric interaction pattern is consistent with experimental evidence for the conserved aminergic Asp116 anchor and with the serotonin-bound 5-HT_1A_ structure [21,22]; it was therefore used as the reference fingerprint for comparison with α,α-trehalose.

Although several residues overlapped with the 5-HT contact set, the α,α-trehalose pose did not reproduce the complete canonical orthosteric fingerprint. Instead, α,α-trehalose was assigned a top-ranked Vina affinity score of −6.3 kcal/mol and adopted a more outward pose near the extracellular entrance of the binding cavity. The predicted contact set included Ala93, Tyr96, Gln97, Phe112, Ile113, Asp116, Cys187, Thr188, Ile189, Lys191, Thr379, Ala383, Asn386, Trp387, and Tyr390. Conventional hydrogen bonds were predicted with Thr188, Asn386, and Tyr390, whereas the remaining residues contributed mainly van der Waals or other weak non-covalent interactions (Figure 9).

In contrast to the deep orthosteric pose of 5-HT, α,α-trehalose was positioned closer to an extracellular-facing peripheral region, as supported by its outward orientation and contacts with Thr379 and Ala383. Key residues contacted by 5-HT deeper within the orthosteric cavity—including Asp192, Tyr195, Thr196, Ser199, Thr200, Ala203, Trp358, Phe362, and Pro369—were absent from the α,α-trehalose contact profile. This noncanonical pose is consistent with the bulky and highly hydrophilic nature of α,α-trehalose, suggesting that it reflects surface-accessible polar contacts rather than productive orthosteric engagement. These results should be interpreted as a structural hypothesis and do not demonstrate specific binding, allosteric modulation, or pharmacological activity at the 5-HT_1A_ receptor. Consequently, while no direct pharmacological interaction of α,α-trehalose at the 5-HT_1A_ receptor is implied, a possible indirect or external influence cannot be entirely ruled out.

#### 2.2.5. In Vivo Magnetic Resonance Imaging (MRI)

In vivo *diffusion tensor imaging analysis:* Figure 10A,B show delimited regions of both hemispheres in a coronal section of the brain of mice evaluated 30 min after α,α-trehalose administration at a dose of 100 mg/kg, i.p. The regions of interest were manually outlined. The values reported for each brain structure represent the average of both sides. No significant changes were found in any of the evaluated regions when analyzing the apparent diffusion coefficient, such as in the Dorsal Hippocampus (DH) (Figure 10C), the Thalamic Reticular Nucleus (TRN) (Figure 10D), and the Arcuate Nucleus (AN) (Figure 10E). In contrast, the TRN (Figure 10D) and the AN (Figure 10E) showed a significant increase in the fractional anisotropy (FA) map (↑21%, *p* < 0.05 and ↑64%, *p* < 0.01, respectively), while a trend towards an increase in FA was noticed in the DH (Figure 10C).

## 3. Discussion

Here, we show the chemical analysis and preclinical pharmacological in vivo, in situ, and in silico evaluation of α,α-trehalose—a disaccharide identified and purified as a bioactive metabolite of *Psilocybe cubensis*, distinct from the known tryptamine-type alkaloids also found in the polar extracts of this mushroom. These results provide scientific evidence supporting the biological properties of this disaccharide in the CNS, demonstrating its ability to induce significant changes in brain activity and to produce anxiolytic- and antidepressant-like behavioral responses in murine experimental models, ruling out a direct interaction on the 5-HT_1A_ serotonin receptor.

Mexican indigenous peoples have used *Psilocybe* mushrooms in religious ceremonies to prevent health or mental problems. These natural products are known as neurotropic, psychedelic, psychoactive, psychotropic, entheogenic, and hallucinogenic, among others, and it has been suggested that strict rules should be followed to avoid their abuse, mainly through recreational use [13]. It has been stated that the effects of mushrooms are significantly less dangerous in human participants reporting previous use of magic mushrooms than mushroom-naïve respondents [23]. *Psilocybe cubensis* was chosen as the fungal material for preliminary pharmacological investigation since it is the most well-known species, and it is used all over the world [24]. It grows easily and is considered moderately potent compared to other active species, containing up to 1–2% (dry weight) of tryptamine alkaloids such as psilocybin, psilocin, and baeocystin [25].

A few cases of preclinical treatment with mushrooms have been reported to be efficacious for anxiety and/or depression. In a preliminary study, anxiolytic- and antidepressant-like activities, without sedative effects, were confirmed using enteral (p.o.) or parenteral (i.p.) administration of the whole *Psilocybe cubensis* mushroom in different experimental models in mice, yielding responses that resembled those of known anxiolytic and/or antidepressant drugs [18]. Recent preclinical studies have shown that *Hericium erinaceus* produces dose-dependent antidepressant-like effects through the modulation of different neurotransmitter systems [26]. Likewise, a formulation containing *Ganoderma lucidum* (400 mg/kg) combined with *Rosa roxburghii* (800 mg/kg) prevented depressive-like behavior in mice by increasing serotonin levels [27].

It should be noted that the most extensive evidence in the literature is available for alkaloids of the *Psilocybe* species [28], whose physiological, neuroendocrine, and psychological effects have been widely documented. These effects include alterations in cognition, sensory processing, and time perception, which have been associated with their potential to prevent or treat neurological and psychological disorders [29]. However, other bioactive metabolites in addition to alkaloids are considered to have great brain health properties, such as α,α-trehalose—a disaccharide that produces CNS activity by acting in a possible synergistic manner to enhance the potential anxiolytic and antidepressant-like effects of this mushroom. This information agrees with preliminary data using an alkaloid extract of *Psilocybe cubensis* in mice [30], where anxiolytic properties were found in experimental Alzheimer’s disease after treatment with 4% α,α-trehalose for 14 days [31] or in a model of Parkinson’s disease using 2% α,α-trehalose for 6 months [32]. In this regard, increasing evidence indicates that the antidepressant potential of medicinal mushrooms is not restricted to the alkaloids of *Psilocybe* species.

It is known that α,α-trehalose is present only in non-mammalian species, such as bacteria, yeast, fungi, insects, invertebrates, and even plants, to protect their cells against environmental stress [33]. α,α-Trehalose possesses greater structural flexibility due to its 1,1-glycosidic linkage, a characteristic that favors its interaction with polar groups, including alkaloids such as psilocybin. According to a previous report [34], α,α-trehalose concentration is related to the survival capacity of organisms, and in *Psilocybe cubensis*, it represents the major metabolite [17]. Furthermore, its function as a natural stabilizer against stressful conditions, such as extreme temperatures, dehydration, and nutrient limitation, along with its industrial application as an enzyme protectant and preservative [35], suggests this disaccharide might promote the preservation of primary and secondary metabolites. In this context, it is reasonable to propose that the metabolomic profile of fresh *Psilocybe cubensis* could differ from that observed in dehydrated samples, depending on α,α-trehalose’s ability to preserve certain metabolites, which should be confirmed in future studies.

As the presence of α,α-trehalose in mushrooms and plants plays a crucial role in preserving enzymes and protecting membranes during dehydration, it allows the wild fungus to survive in more challenging environments [36]. In a similar manner, it has been reported that most species of *Selaginella* can survive severe drought conditions due to the presence of α,α-trehalose as a unique disaccharide that maintains the plant’s structural stability and allows it to recover upon rehydration [37]. In addition, α,α-trehalose has gained relevance due to its remarkable neuroprotective properties in models of neurodegenerative and neuropsychiatric diseases [33], which are attributed to its high chemical stability and bioactive capabilities in osmoprotection, anhydrobiosis, cryoprotection, antioxidant activity, and autophagy activation [31,38,39]. α,α-Trehalose has been considered a potential neuroprotective compound [40,41] that reduces microglial activation and reverses cytoskeletal disruption in astrocytes, thereby diminishing the glial inflammatory response and restoring the normal morphology of astrocytes [42].

The acute effects of α,α-trehalose could be partly explained by its antioxidant properties, the attenuation of neuroinflammation, and the preservation of serotonin levels, which, although not explored in this research, have been reported to reduce stress-induced reactive oxygen species (ROS) during the FST in the cortex and hippocampus and prevent neuronal damage [31,39,40,43]. The reduction in pro-inflammatory cytokine levels attenuates the activation of indoleamine 2,3-dioxygenase, thereby preventing serotonin depletion [44], which may contribute to the observed increase in swimming behavior. Together, these studies position α,α-trehalose as a key metabolite that may complement the therapeutic activity of classical alkaloids such as psilocybin and psilocin, thereby contributing to the effects of *Psilocybe cubensis* on mental health. Alterations in platelet morphology and activity, as well as in nitric oxide synthesis, and/or 5-HT concentrations, were previously reported in rats exposed to the FST to induce depressive-like behavior, with these changes being reversed by the antidepressant fluoxetine [45]. A similar pattern was also observed in platelets from patients with a diagnosis of depression [46,47]. α,α-Trehalose has been shown to ameliorate mild emotional and social deficits by reducing anxiety-like, social, and predator-avoidance behaviors in the mutant zebrafish cln8−/− [48]. In addition, a significant increase in doublecortin and synaptophysin levels in the cerebral cortex, as well as in progranulin protein expression in the cortex and hippocampus, was observed in mice treated with α,α-trehalose. These changes were associated with improved cognitive performance in the Tg2576 mouse model of Alzheimer’s disease, as assessed using the Morris water maze test [49].

Regarding the ECoG findings, α,α-trehalose enhanced cortical oscillatory activity rather than causing a generalized suppression of neuronal activity. These changes may underlie its observed anxiolytic and antidepressant-like effects and are consistent with its previously reported neuroprotective and autophagy-related actions. These effects may involve not only the promotion of neuronal protein homeostasis but also changes in electrophysiological activity associated with more synchronized cortical states. In murine models, chronic administration of α,α-trehalose increases delta-band power and improves recovery from sleep deprivation, suggesting an enhanced slow-wave oscillatory activity, associated with sleep homeostasis [50]. This effect may be interpreted not as a direct hypnotic action, but rather as a facilitation of homeostatic processes related to neuronal integrity and intracellular clearance, which are closely coupled to slow-wave dynamics. The ECoG spectral analysis showed that α,α-trehalose alone induced changes in cortical oscillatory activity, characterized by increased power in the delta (1–4 Hz), sigma (10–15 Hz), and beta (13–30 Hz) bands. Given the short duration of the recording period and the absence of polysomnographic measurements—a study limitation regarding the identification of sedative activity—these findings are best interpreted as an increase in cortical synchronization rather than as evidence of a sleep state. The simultaneous increase in power across multiple frequency bands indicates a spectral organization of cortical activity, reflecting modulation of thalamocortical networks involved in the generation of slow oscillations [51]. In particular, the increase in delta- and sigma-band power is consistent with more synchronized cortical states, while the modulation of beta-band activity indicates that α,α-trehalose does not simply suppress cortical excitability but rather promotes a more structured oscillatory pattern. Importantly, these electrophysiological changes are consistent with the anxiolytic and antidepressant-like effects observed in behavioral tests, suggesting a functional association between the α,α-trehalose-induced modulation of cortical activity and affective regulation. Although a direct causal relationship cannot be established, changes in low-frequency cortical activity have been associated with changes in emotional processing and stress-related neuronal activity [52,53], supporting the possibility that the observed oscillatory modifications may reflect brain states associated with greater emotional stability. Interestingly, the effects observed with α,α-trehalose were potentiated in the presence of the 5-HT_1A_ receptor antagonist WAY-100635, suggesting that α,α-trehalose may modulate the baseline response of cortical circuits rather than act as a direct depressant. Previous studies have shown that WAY-100635 potentiates the increase in extracellular serotonin produced by antidepressants and reinforces presynaptic serotonergic function under conditions where neuronal activity is pharmacologically suppressed [54,55]. Given the well-established role of serotonergic signaling in regulating mood and cortical excitability [52], this interaction raises the possibility that α,α-trehalose modifies the functional state of cortical networks, thereby increasing their sensitivity, at least in part, to serotonergic modulation. Further studies are necessary to clarify the mechanisms underlying these effects.

The ECoG analysis supports the influence of α,α-trehalose at the CNS level, not as a 5-HT_1A_ ligand, but as a possible membrane or protein stabilizer, as reported in the literature [35]. In this regard, the exploratory docking analysis assessed the predicted binding behavior of α,α-trehalose to the 5-HT_1A_ receptor (PDB: 7E2Y) and compared it with that of 5-HT, used as an orthosteric structural reference, and revealed qualitatively distinct contact fingerprints between these two compounds. Although the docking algorithm accommodated α,α-trehalose within the ligand-binding cavity, the physicochemical plausibility of a productive orthosteric binding mode remains limited. Compared with 5-HT, α,α-trehalose is larger, more flexible, highly polar, and lacks the protonated amine that forms a conserved ionic interaction with Asp116 in aminergic receptor ligands [21,22]. Its multiple hydroxyl groups are expected to be strongly solvated in the aqueous extracellular environment, making transfer into a relatively buried transmembrane cavity energetically demanding. Standard docking scores may overestimate such poses because they do not fully capture ligand desolvation, water displacement, conformational entropy, receptor flexibility, or the effects of the membrane [56]. Therefore, the present results do not provide evidence of direct, specific binding of α,α-trehalose to 5-HT_1A_, although they cannot completely exclude a weak or noncanonical interaction. As previously mentioned, the effects of α,α-trehalose have instead been associated with autophagy modulation and the stabilization of proteins and membranes under stress or dehydration conditions. The potentiated depressant response observed after 5-HT_1A_ receptor blockade may indicate the involvement of serotonergic signaling in the biological response, but it does not establish direct receptor engagement by α,α-trehalose. It is important to note that these outcomes allow the formulation of a structural hypothesis without constituting evidence of receptor activation, functional affinity, competitive binding, or allosteric modulation. This would require complementary molecular dynamics simulations (MDSs) to provide additional information on the temporal stability of the ligand–receptor complex, conformational changes, and the potential impact of ligand binding at the receptor interaction site. This represents a limitation of the present analysis based on bioinformatic tools. However, preliminary MDS analysis of α,α-trehalose revealed that dynamic movement around the glycosidic bond is slow, allowing the disaccharide to maintain its conformation without direct interaction with biological macromolecules [57].

According to the NMR data, it is known that FA and the ADC values obtained using DTI express the preferred direction of water molecule movement within tissues, where high values indicate preserved organization and integrity of cerebral white matter [23]. Marked directional diffusion is reflected by a high degree of anisotropy, which ranges from 0 (identical diffusion in all directions, characteristic of cerebrospinal fluid) to 1 (strictly unidirectional diffusion, theoretical). A decrease in these parameters may indicate demyelination, diffuse axonal damage following traumatic brain injury, or microstructural alterations associated with neurodegenerative diseases. This preliminary NMR results refers that water diffusion at CNS is promoted in the presence of α,α-trehalose involving brain regions, such as the AN and TRN observed in the first 30 min after its administration. At this time and dosage of 100 mg/kg, i.p., we also observed a depressant neuronal activity registered in ECoG in mice that showed anxiolytic and antidepressant-like behavioral responses supporting the CNS activity of α,α-trehalose to improve mental health.

To our knowledge, no experimental evidence has demonstrated direct binding of α,α-trehalose on specific receptors, which is consistent not only with the non-orthosteric predicted pose observed in the molecular docking analysis but also with the physicochemical profile of this disaccharide as a highly polar, non-reducing sugar. Its neuroprotective effects have been associated with autophagy modulation, protein/membrane stabilization, and attenuation of oxidative or stress-related signaling pathways rather than canonical serotonin receptor engagement [58]. The predicted conformation reflects accessible polar contacts on the receptor surface rather than a productive orthosteric engagement, suggesting that α,α-trehalose activity could not be mediated by direct interaction at 5-HT_1A_ receptors. An enhanced depressant response in ECoG recordings associated with 5-HT_1A_ serotonin receptor blockade suggests that indirect mechanisms could influence the effects of α,α-trehalose on brain activity. This interaction warrants further investigation, particularly in relation to the indole ligands of this receptor, given its potential physiological and therapeutic relevance to mental health.

The limitations of this preliminary molecular docking analysis should be noted. First, molecular docking provides static predictions that are useful for identifying plausible binding poses and interaction patterns, although it does not directly characterize binding kinetics, conformational selection, receptor activation, or signaling efficacy [58]. Second, the docking scores reported here span a narrow range (−6.1 to −6.3 kcal/mol), within which differences cannot be reliably distinguished by the Vina scoring function as evidence of differential affinity [57,59]. Third, the double-docking model imposes rigid occupancy by serotonin and cannot capture the dynamic, competitive nature of ligand binding in a membrane environment. In addition, factors such as α,α-trehalose desolvation, explicit water networks, membrane lipids, receptor flexibility, and conformational entropy are only partially represented in the current model. Future studies incorporating molecular dynamics simulations, MM-GBSA rescoring, explicit membrane/water conditions, and experimental validation through radioligand displacement and functional signaling assays will be necessary to test the structural hypotheses generated here, as well as the biological effects of α,α-trehalose on this and other receptors. Pharmacokinetic evaluation to characterize the temporal course of concentrations and distribution of α,α-trehalose is another limitation of the research.

## 4. Materials and Methods

### 4.1. Fungal Material

*Psilocybe cubensis* (Earle) Singer was collected in August 2022 in the municipality of Xochicoatlán, Hidalgo, Mexico, and was identified by Dr. Leticia Romero Bautista from the Laboratory of Integral Mycology, Department of Biology, Universidad Autónoma del Estado de Hidalgo. A specimen has been deposited in the “Gastón Guzmán Huerta” Herbarium of the Escuela Nacional de Ciencias Biológicas, Instituto Politécnico Nacional, under reference number 121805.

### 4.2. General Information on Chemical Analysis

Melting points were measured using a Fisher-Johns apparatus (Pittsburgh, PA, USA) and are uncorrected. Infrared spectra were obtained using a PerkinElmer FT-IR System 2000 spectrophotometer (PerkinElmer, Walthan, MA, USA) with potassium bromide (KBr) tablets. ^1^H and ^13^C NMR spectra were obtained using a Bruker Ascend instrument (Billerica, MA, USA) (400 MHz for ^1^H; 100 MHz for ^13^C) in D_2_O solutions. Optical rotation was recorded using a PerkinElmer 341 polarimeter (Walthan, MA, USA). An authentic sample of D-(+)-α,α-trehalose was acquired from Sigma (St. Louis, MO, USA, CAS 6138-23-4). Methanol (CAS 67-56-1) and HPLC-grade hexane (CAS 110-54-3), as well as glacial acetic acid (CAS 64-19-7), were purchased from Fermont (Monterrey, Mexico). Microcrystalline cellulose for column chromatography (CAS No. 9004-34-6) was purchased from Merck (Mexico City, Mexico), and 1-butanol (CAS No. 71-36-3; JT Baker (Monterrey, Mexico) was procured from El Crisol (Mexico City, Mexico).

### 4.3. Preparation of the MeOH Extract and α,α-Trehalose

The MeOH extract of *Psilocybe cubensis* was obtained according to a previously described procedure [18]. Thus, the fungal material (68.10 g) yielded 11.85 g (17.41%) of MeOH extract. A portion of this extract (10 g) was dissolved in distilled water (500 mL) and extracted twice with an equal volume of HPLC-grade hexane. The organic phase (OF) was dried over anhydrous sodium sulfate (Na_2_SO_4_) and filtered. Both phases were concentrated under reduced pressure using a rotary evaporator, yielding the OF (0.650 g) and the aqueous fraction (AQ, 5.84 g).

The AQ (2.50 g) was subjected to CC using microcrystalline cellulose (134 g) as the stationary phase and 1-butanol saturated with H_2_O (1.5 L) and distilled water (1 L) as the mobile phase. Thirty-six fractions of 20 mL each were collected. In fraction 31 (0.496 g), the presence of α,α-trehalose was evident, and a portion (0.350 g) was further purified by CC using microcrystalline cellulose (49 g), 1-butanol saturated with H_2_O (180 mL), 5% glacial acetic acid (CH_3_COOH; 100 mL), and distilled water (70 mL), yielding 27 fractions of 10 mL each (Figure 1). α,α-Trehalose was obtained as colorless crystals in fraction 15 (106.4 mg, 30.3% and 0.16% based on the initial fungal material), which were mechanically separated and washed with hexane to provide colorless prismatic crystals with a m.p. of 96–99 °C (m.p. 95–97 °C [60]). This sample showed ${\left[\alpha \right]}_{D}^{20}$ + 165 (*c* 0.02, H_2_O) (${\left[\alpha \right]}_{D}^{20}$ + 169, [60]); IR *ν*_max_: 3349, 2907, 1457, 1000; ^1^H–NMR (400 MHz, D_2_O, 28 °C) δ 5.21 (2H, d, *J* = 3.8 Hz, H-1), 3.88 (2H, dd, *J* = 12.1, 1.8 Hz, H-6a), 3.87 (2H, t, *J* = 9.9 Hz, H-3), 3.85 (2H, ddd, *J* = 9.4, 5.0, 1.8 Hz, H-5), 3.77 (2H, dd, *J* = 12.1, 5.0 Hz, H-6b), 3.66 (2H, dd, *J* = 9.9, 3.8 Hz, H-2), 3.46 (2H, t, *J* = 9.4 Hz, H-4), ^13^C NMR (100 MHz, D_2_O, 28 °C) δ 93.2 (C-1), 72.5 (C-3), 72.1 (C-5), 71.0 (C-2), 69.7 (C-4), 60.5 (C-6).

### 4.4. Single Crystal X-Ray Diffraction Analysis of α,α-Trehalose

The data were collected on an Agilent Xcalibur Atlas Gemini diffractometer using an enhanced MoKα X-ray source (λ = 0.71073 Å) at 293(2) K in ω-scan mode. Unit cell parameters were refined using 3644 machine-detected reflections with the CrysAlisPro software v.11.171.41.112a, Agilent Technologies (Santa Clara, CA, USA). Crystal data were as follows: C_12_H_22_O_11_·2(H_2_O), crystal size 0.5 × 0.4 × 0.4 mm^3^, M = 378.33, orthorhombic, space group *P*2_1_2_1_2_1_, *a* = 7.5847(2) Å, *b* = 12.2168(3) Å, *c* = 17.8625(4) Å, V = 1655.15(7) Å^3^, Z = 4, Z’ = 1, ρ = 1.518 mg/mm^3^, μ(MoKα) = 0.139 mm^−1^, total reflections = 56,580, unique reflections = 3644 (*R*_int_ = 0.0720), and observed reflections = 3644. Data were processed using Olex2 v1.5 software [61]; the structure was solved by intrinsic phasing using the ShelXT v(2014/3) program [62]. Structural refinement was performed using the ShelXL v(2018/3) program [63]. Non-hydrogen atoms were refined anisotropically, and most hydrogen atoms were refined at geometrically idealized positions on their parent atoms. The O–H hydrogen atoms were observed in the difference maps. Final R indices were [I > 2σ(I)] R_1_ = 0.0295 and wR_2_ = 0.0785. The largest difference peak and hole were 0.20 and −0.15 e.Å^−3,^ respectively. The Flack parameter is 0.5(9) and the Hooft parameter is 0.6(2); these data are far from the expected values because of the use of Mo radiation; however, the enantiomer observed in this model matched the published one [20].

### 4.5. Pharmacological Evaluation of α,α-Trehalose

#### 4.5.1. Animals

Male *Swiss Webster* mice (25–30 g) aged 8 ± 1 weeks, obtained from the vivarium of the Instituto Nacional de Psiquiatría Ramón de la Fuente Muñiz (INPRFM), were kept at a controlled temperature (22 ± 2 °C) with a 12-h light/dark cycle and free access to food and water. The animals were assigned to groups of at least six individuals. The experimental protocol followed the recommendations of the Norma Official Mexicana (NOM-062-ZOO-1999), the guidelines of the institutional committees such as Biosafety CB005_23 (13 April 2023), CICUAL/02/2024 (18 December 2024), CEI/C/022/2025 (16 June 2025), and the INPRFM research protocol NC25230.0 (19 August 2025).

#### 4.5.2. Drugs and Reagents

Imipramine (IMI, 10 mg/kg, i.p., antidepressant reference drug), buspirone (4 mg/kg, i.p., anxiolytic reference drug), and α,α-trehalose dihydrate were obtained from Sigma-Aldrich (St. Louis, MO, USA). Pure compounds and the reference drugs were freshly prepared on the day of the experiments. Three logarithmically increasing doses were tested in order to use the fewest number of animals to determine if the observed effects are dose-dependent. Doses were selected from previously published reports or preliminary experiments conducted in the laboratory [18,64]. It is considered that observing physiological functions after in vivo administration of α,α-trehalose may be difficult due to its digestion by trehalase [65]. Thus, to reduce this process and increase bioavailability, intraperitoneal administration (i.p.) was used.

#### 4.5.3. Experimental Design

A total number of eighty mice was used in the whole study: forty-two mice in the pharmacological evaluation; thirty mice for ECoG recordings; and eight mice for NMR analysis. For behavioral tests, forty-two mice were divided into groups of *n* = 6 to receive one of the following treatments: (1) Vehicle (distilled water), (2–4) α,α-trehalose (1, 10, and 100 mg/kg, i.p.), and (5 or 6) the reference drug IMI (10 mg/kg, i.p.) or buspirone (4 mg/kg, i.p.). Behavioral responses to determine anxiolytic and/or antidepressant-like activity were evaluated using hole-board and plus-maze tests, complemented by open-field test, as well as the forced swimming test (FST), accompanied by the rota-rod test, respectively. The animals were habituated to the environment for at least three days before the tests were carried out. The treatment groups were compared with mice receiving the vehicle (distilled water, 1 mL/100 g body weight). The effects were evaluated 30 min after treatment administration. Each apparatus was cleaned with a 70% ethanol solution after each individual experiment. In situ electrocorticographic recordings were performed in mice to determine changes in neuronal activity and confirm CNS effects. After a preliminary in silico molecular docking analysis, the participation of the 5-HT_1A_ serotonin receptor was explored in the in vivo and in situ experiments in the presence of (7) WAY-100635—a competitive antagonist (1 mg/kg, i.p.)—administered alone or (8) in combination with α,α-trehalose (100 mg/kg, i.p.).

#### 4.5.4. Anti-Anxiety Behavioral Evaluation of α,α-Trehalose

*The open field test*: Each mouse was placed in an acrylic box divided into 12 squares (6 × 6 cm). The number of squares explored over a period of 2 min was considered a measure of ambulatory activity [66]. In addition, the number of times the mice stood on their two hind limbs, placing their two forelimbs on the cage wall (rearing behavior), was counted for the same 2 min to further assess the escape behavior. A significant reduction in ambulatory activity was associated with a sedative effect, while a reduction in rearing events was considered an anxiolytic-like response [67].

*The elevated plus-maze test*: To corroborate the anxiolytic-like activity of the treatments, the well-known and widely validated elevated plus-maze test was also included in the behavioral evaluation [68]. It consisted of a wooden cross, elevated 50 cm above the base, with two open arms (30 × 5 cm) and two closed arms (30 × 15 × 5 cm), and a central open space (5 × 5 cm) where each mouse was placed. The latency and the number of entries into each closed or open arm, as well as the time spent in each arm and the average time in the open arm, were recorded over a period of 5 min.

#### 4.5.5. Antidepressant Effect of α,α-Trehalose

*Rota-rod test*: The effects of the treatments on motor coordination were analyzed using a Rota-rod treadmill for mice (model 7600, Ugo Basile, Comerio, Italy) 4 cm in diameter. First, animals were trained to remain on the rod for at least 5 min. The mice that completed the preliminary test were selected and grouped into groups of six animals each. Then, 30 min after treatment administration, the number of falls was counted for each mouse during an observation of at least 2 min in the rotating cylinder [67]. This test was performed before evaluating antidepressant activity to confirm the mice’s ability to swim in the Forced Swimming Test (FST).

*Forced Swimming Test (FST)*: Animals were individually placed in a glass cylinder (26 cm high, 12 cm in diameter) filled with 17 cm of tap water (25 ± 2 °C). The FST consisted of two swimming sessions. In the first session, called the pre-test, mice swam for 15 min. Twenty-four hours later, the animals were exposed to a second 5 min session, called the final test. At the end of each session, the mice were removed from the cylinder, towel-dried, and placed in a cage with sawdust under a red-light lamp for 15 min before being returned to their home cages [69]. The test was videotaped and scored by an observer blinded to the treatment.

A time-sampling technique was used to score the presence of the following behaviors: (a) immobility, defined as floating without struggling and making only small movements necessary to keep the head above the surface of the water; (b) swimming, defined as active movements in which the animals moved and dived around the cylinder; or (c) climbing, defined as vigorous movements in which the rats moved their forepaws in and out of the water, generally directed toward the wall of the cylinder. The number of occurrences of each behavior was recorded every 5 s for 5 min.

#### 4.5.6. Electrocorticographic (ECoG) Recording and Spectral Power Analysis

The ECoG power spectrum analysis was used to explore how physiological signals in the mouse brain are modified under experimental conditions of anxiety or depression, with or without pharmacological treatment with α,α-trehalose, and to investigate the potential involvement of the 5-HT_1A_ receptor as a target under these conditions.

*Electrode implantation surgery and ECoG recordings*: Thirty adult male *Swiss Webster* mice (8 weeks old, 28–35 g) were anesthetized with isoflurane (4% induction, 1–2% maintenance) and secured in a stereotaxic frame (Stoelting, Wood Dale, IL, USA). Two stainless-steel screw electrodes were implanted bilaterally over the frontal cortices (AP +2.0 mm; ML 1 mm). A reference electrode was placed over the cerebellum (AP +5 mm; ML 0) [70]. All electrodes were soldered to a miniature connector and fixed to the skull using dental acrylic cement. Following surgery, the animals were allowed to recover for at least one week before electrophysiological recordings.

After recovery, the animals were placed in a sound-insulated chamber and allowed to habituate for 20 min before recordings. ECoG activity was then recorded continuously for at least 40 min, including a 10 min baseline period followed by 30 min activity evaluation after treatment administration. The groups consisted of (1) vehicle, (2) reference drug buspirone (4 mg/kg, i.p.), (3) antagonist of the 5-HT_1A_ serotonin receptor WAY-100635 (WAY, 1 mg/kg, i.p.), (4) the identified bioactive metabolite α,α-trehalose (100 mg/kg, i.p.), or (5) a combination of WAY (1 mg/kg, i.p.) + α,α-trehalose (100 mg/kg, i.p.) (*n* = 6 per group). ECoG signals from both frontal cortices were amplified and bandpass filtered (1–70 Hz) using an electroencephalograph GRASS Model 8-18D (GRASS Instrument Co., Quincy, MA, USA) and digitized at a sampling rate of 500 Hz.

*ECoG spectral power analysis*: The effects of each treatment on ECoG activity recorded from the left frontal cortex were analyzed using spectral power analysis. Power spectra were computed using custom-made routines developed in MATLAB (R2020a, The MathWorks Inc., Natick, MA, USA), applying the fast Fourier transform (FFT) to signals within the 1–50 Hz frequency range. FFT was applied to five artifact-free 60 s segments obtained during the baseline period and to additional 60 s segments collected at 30–35 min post-treatment. Absolute spectral power was calculated for the delta (1–4 Hz), theta (4–10 Hz), sigma (10–15 Hz), beta (15–30 Hz), and gamma (30–50 Hz) frequency bands for each animal, and the numerical values were then normalized as a percentage of change from baseline.

#### 4.5.7. Docking Analysis of α,α-Trehalose

*Ligand and Receptor Preparation*: α,α-trehalose (α-D-glucopyranosyl-α-D-glucopyranoside) was prepared as the three-dimensional ligand structure and subjected to energy minimization using the Merck Molecular Force Field (MMFF94) in Avogadro to optimize its geometry (Avogadro Chemistry, Pittsburgh, PA, USA; Merck Research Laboratories, Boston, MA, USA) [71]. Serotonin (5-hydroxytryptamine, 5-HT) was included as the endogenous orthosteric reference ligand. The receptor structure for 5-HT_1A_ (5-HT receptor 1A) was obtained from the Protein Data Bank (PDB) (PDB ID: 7E2Y, https://www.rcsb.org/; accessed on 20 February 2025) at the highest available resolution. [21]. Receptor preparation involved the removal of water molecules and heteroatoms, the addition of polar hydrogens, and the assignment of Gasteiger partial charges using AutoDock v.4.2.6. Tools [72].

*Molecular Docking Simulations*: Molecular docking was performed using AutoDock Vina v.1.2.x to compare the predicted poses and interaction profiles of α,α-trehalose and 5-HT at the 5-HT_1A_ receptor. Grid boxes were centered on the receptor binding cavity and defined to encompass the ligand-binding region within the transmembrane helical bundle. The exhaustiveness parameter was set to 10 to ensure thorough exploration of the binding pocket. For each ligand–receptor pair, the five best-ranked poses were generated, and the pose with the most favorable AutoDock Vina score was selected for interaction analysis. The reported values are Vina scoring estimates (kcal/mol), not experimentally determined binding free energies. Post-docking analyses were conducted using UCSF ChimeraX and BIOVIA Discovery Studio Visualizer to examine predicted hydrogen bonds, van der Waals contacts, hydrophobic contacts, and aromatic interactions [73,74].

Two docking experiments were performed: (a) serotonin docked to the unoccupied receptor (5-HT-5-HT_1A_), used as a positive orthosteric reference; and (b) α,α-trehalose docked to the unoccupied receptor (α,α-trehalose-5-HT_1A_).

*Interaction analysis*: Docking poses were visualized and analyzed using UCSF ChimeraX and BIOVIA Discovery Studio Visualizer [73,74]. Predicted non-covalent contacts, including conventional hydrogen bonds, van der Waals contacts, hydrophobic interactions, aromatic interactions, and electrostatic contacts, were identified for each complex. Residue overlap with the 5-HT contact set was assessed qualitatively, with particular attention to residues within the established aminergic orthosteric pocket, including the conserved Asp116 anchor and aromatic residues surrounding the ligand-binding cavity [21,22]. Vina scores were used only to rank poses within this exploratory workflow and were not interpreted as direct measures of experimental affinity or ligand efficacy.

#### 4.5.8. In Vivo Magnetic Resonance Imaging (MRI) and Imaging Data Processing

Eight mice, divided into groups of four per cage, were taken to the assigned room with controlled conditions (12-h light/dark cycle, 20 ± 2 °C, 50–60% relative humidity) and free access to food and water for five days before the first scan. Preclinical neuroimaging studies reveal that certain inhaled anesthetics can cause transient brain alterations; to avoid changes in diffusion metrics in rodents due to the presence of the anesthetic, the animals evaluated were subjected to the same imaging protocol and inhaled anesthesia regimen to ensure a uniform impact on the quantification of the FA or ADC, thus making them directly comparable.

The acquisition protocol was carried out at the National Laboratory for MRI using a 7 T scanner (Bruker Pharmascan70/16US, Ettlinglen, Germany). In vivo diffusion tensor images (DTI) were acquired using a CryoProbe transmit/receive surface coil (Bruker, Ettlingen, Germany). The eight mice were scanned two days before and 30 min after α,α-trehalose administration. The animals were anesthetized with a 3% sevoflurane/oxygen mixture and a 2.5% mixture to maintain anesthesia during imaging. Body temperature was maintained using a warm water circulation system. Vital signs were continuously monitored, and anesthesia was discontinued at the end of the imaging session.

The DTI datasets were acquired using a sequence of echoplanar images with the following parameters: repetition time = 2000 ms, echo time = 22.05 ms, number of averages = 2, slice thickness = 750 µm, field of view = 20 × 17.88 mm^2^, matrix = 134 × 120, yielding an in-plane resolution = 149 × 149 µm^2^, and scan time = 27 min. The diffusion-weighted images (DWI) were acquired in 64 unique directions (δ/Δ = 3.3/10 ms), each with three different b-values (650, 1250, and 3000 s/mm^2^).

Briefly, DWI data sets were denoised, and linear transformations were used to correct for motion and eddy-current-induced distortions. The DTI was estimated using the MRtrix 3.0 software. Then, quantitative maps of FA and ADC were created. Regions of interest were manually drawn, outlining the DH, the TRN, and the AN.

### 4.6. Statistical Analysis

Data are expressed as the mean ± S.E.M. of at least 6 mice. Statistical differences were evaluated by one-way analysis of variance (ANOVA) followed by Dunnett’s test for comparisons versus vehicle. Comparisons of absolute spectral power between treatments were performed using one-way ANOVA followed by Tamhane’s T2 post hoc test when appropriate. Before ANOVA, data normality was assessed using the Shapiro–Wilk test, and the homogeneity of variances was evaluated using Bartlett’s test. For potentiation analyses, the theoretical sum was calculated as the expected additive response derived from the individual effects of WAY and α,α-trehalose. This theoretical value was compared with the observed combined effect using Student’s *t*-test. This test was also applied in some cases for comparison between groups. Regarding the imaging data, since no statistical asymmetries were observed following analysis using the paired-sample *t*-test, the average of the left and right regions of interest was used for the values of each structure. All statistical analyses were conducted using GraphPad Prism (version 8.0, GraphPad Software, Inc., La Jolla, CA, USA). Statistical significance was set at *p* < 0.05.

## 5. Conclusions

In conclusion, the mushroom *Psilocybe cubensis,* native to Hidalgo, Mexico, was found to contain the disaccharide α,α-trehalose as bioactive compound after its isolation by chromatographic methods and characterization based on physical and spectroscopic data, including X-ray diffraction analysis. This disaccharide showed significant anxiolytic and antidepressant-like activities in mice, associated with changes in brain activity observed within the first 30 min of its administration in ECoG recordings and in cellular rearrangement, promoting order in the movement of water molecules evaluated by MRI technique, which suggests its likely passage through the blood–brain barrier by a possible facilitated transport mechanism that was not explored in this study. Its presence modified neuronal activity under a depressant condition, which was significantly increased in a non-neurotoxic and potentiated manner when the 5-HT_1A_ serotonin receptor was inhibited, proposing a likely peripheral stabilization of this receptor, as predicted by a preliminary molecular docking analysis but without possibility to act in a direct interaction. It is important to recognize that primary metabolites are considered essential compounds for the basic physiological mechanisms of living organisms, playing a crucial role in their growth and development; however, they can also have a relevant impact on human health, as is already known for several secondary metabolites, which confer multiple beneficial properties on *Psilocybe cubensis* mushrooms as a result of a synergistic action among their multiple bioactive components. These findings expand the knowledge of the chemical composition and therapeutic potential of *Psilocybe cubensis* as a natural product within Mexican Traditional Medicine and as a potential source of drugs for the treatment of affective disorders. The lack of pharmacokinetic analysis to determine the absorption, distribution, and elimination parameters following administration of this disaccharide to promote changes in the CNS represents a limitation of the study. Although the findings are not entirely conclusive, the compound is of interest for future research due to its neuroprotective properties. It is worth noting that the indirect and peripheral mechanisms that induce neuroprotective signals to the brain via the gut–brain axis are other possibilities of interest for future studies on α,α-trehalose.

## Figures and Tables

**Figure 1:**
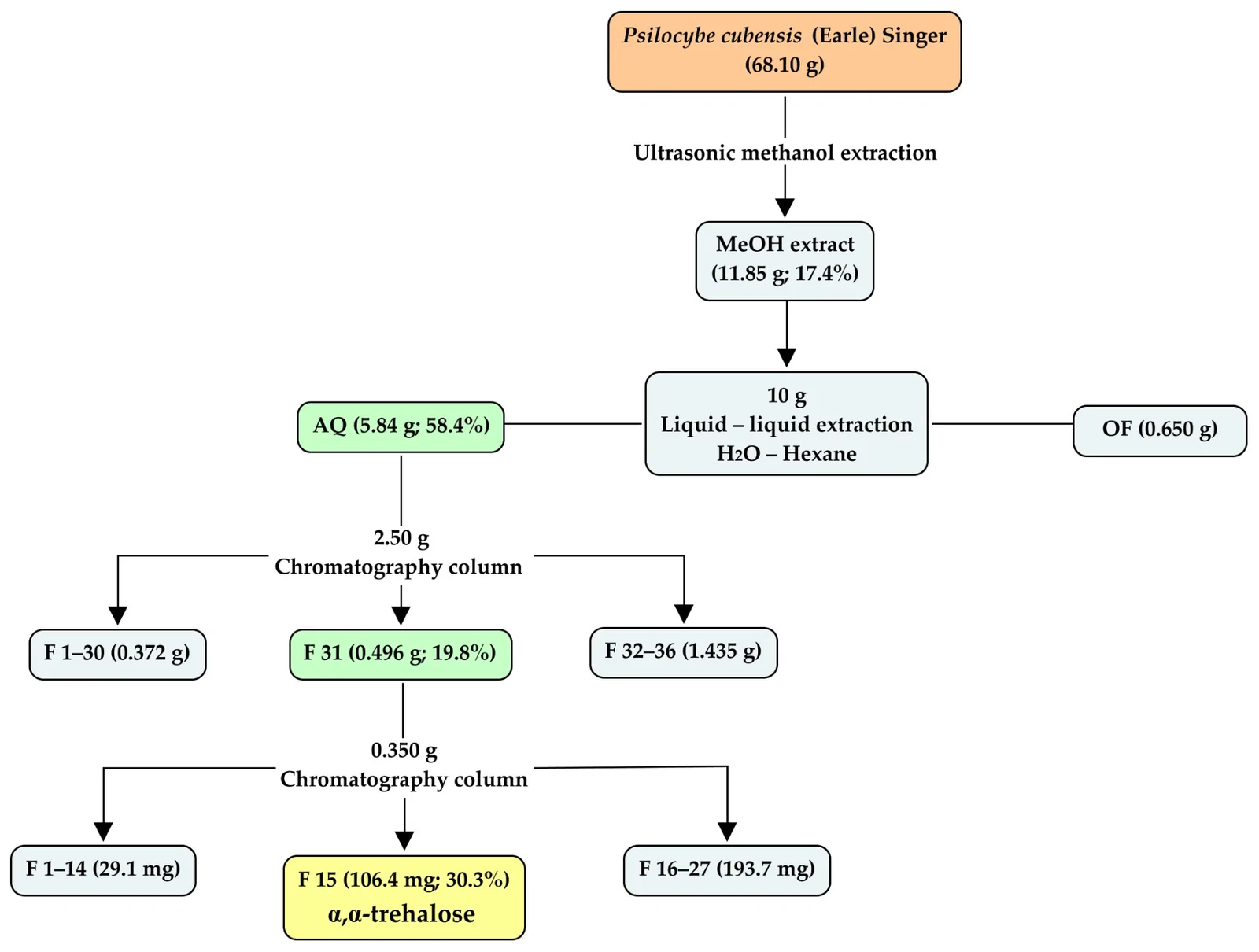
Chemical fractionation of the methanol extract obtained from whole *Psilocybe cubensis* mushrooms to obtain α,α-trehalose. Methanol: MeOH; Aqueous Fraction: AQ; Organic Fraction: OF; Fraction: F.

**Figure 2:**
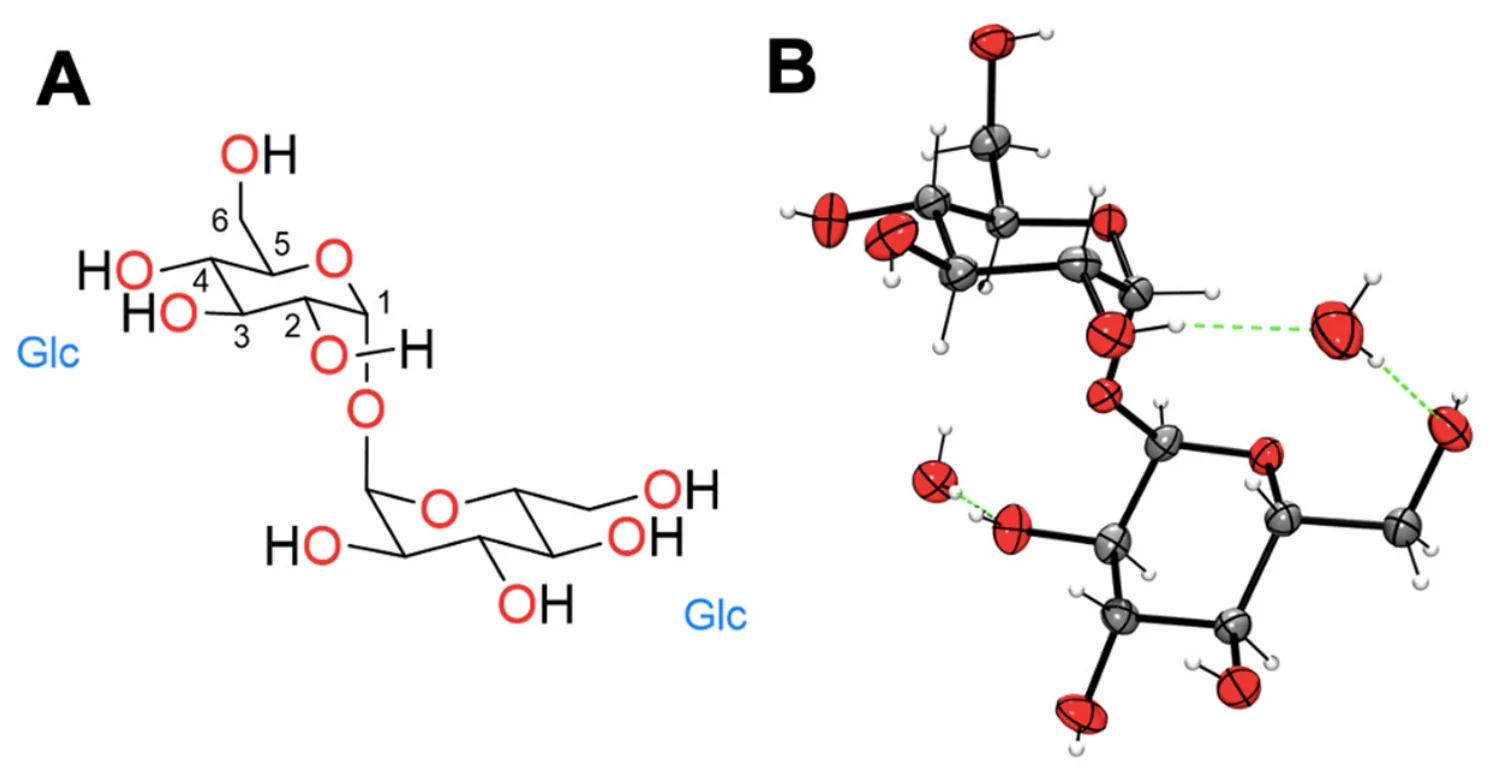
Chemical (**A**) and X-ray (**B**) structures of α,α-trehalose.

**Figure 3:**
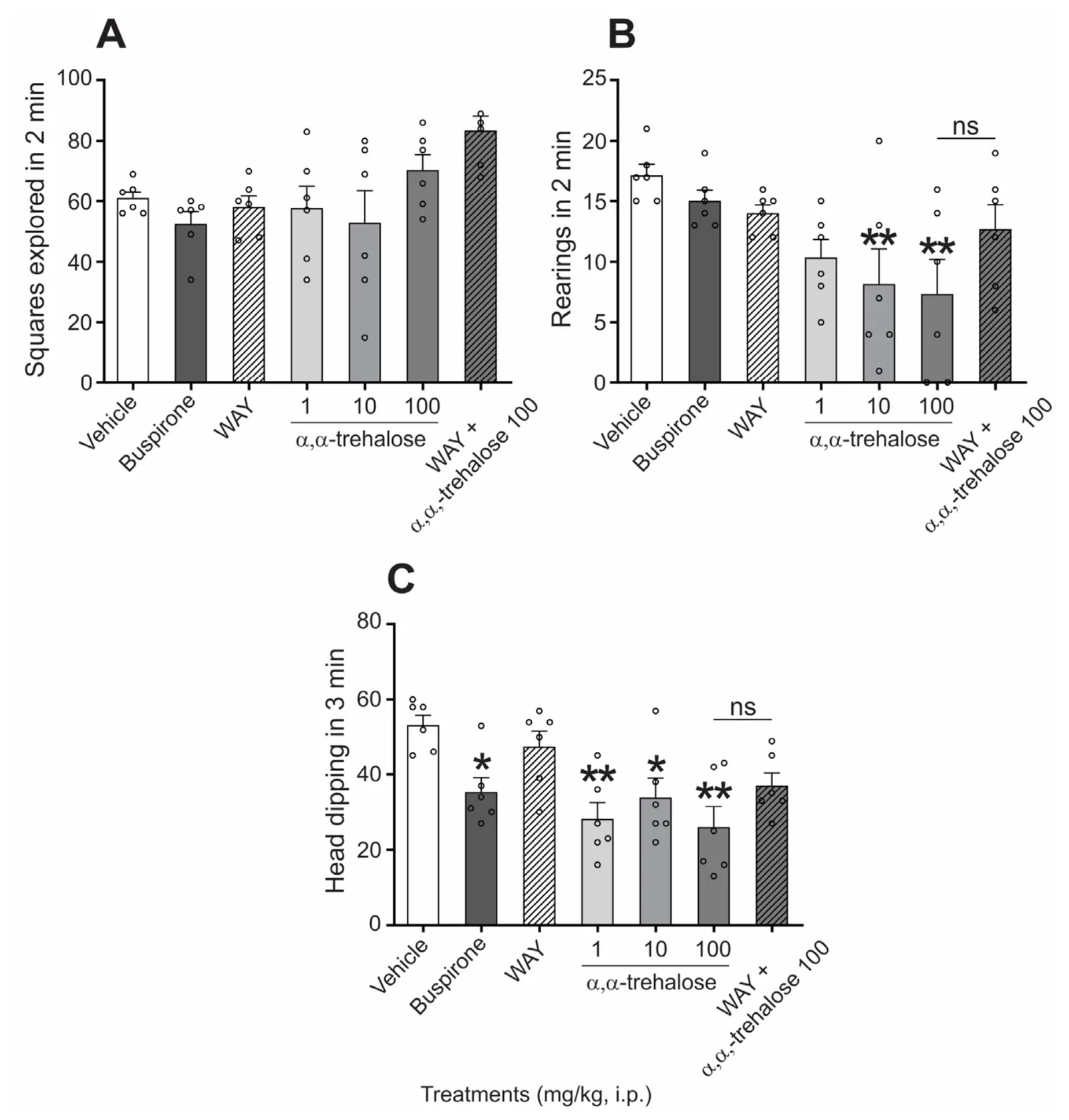
Effects of α,α-trehalose (1, 10 and 100 mg/kg, i.p.) and the reference anxiolytic drug buspirone (4 mg/kg, i.p.), as well as the presence of the antagonist of the 5-HT_1A_ serotonin receptor WAY-100635 (WAY, 1 mg/kg, i.p.) alone and combined with α,α-trehalose (100 mg/kg, i.p.) to evaluate (**A**) squares explored and (**B**) rearing behavior in the open-field test, and (**C**) head-dipping in the hole-board test. Bars represent the mean ± S.E.M., *n* = 6 per group. One-way analysis of variance (ANOVA) followed by Dunnett’s test, * *p* < 0.05 and ** *p* < 0.01. ns: no significant.

**Figure 4:**
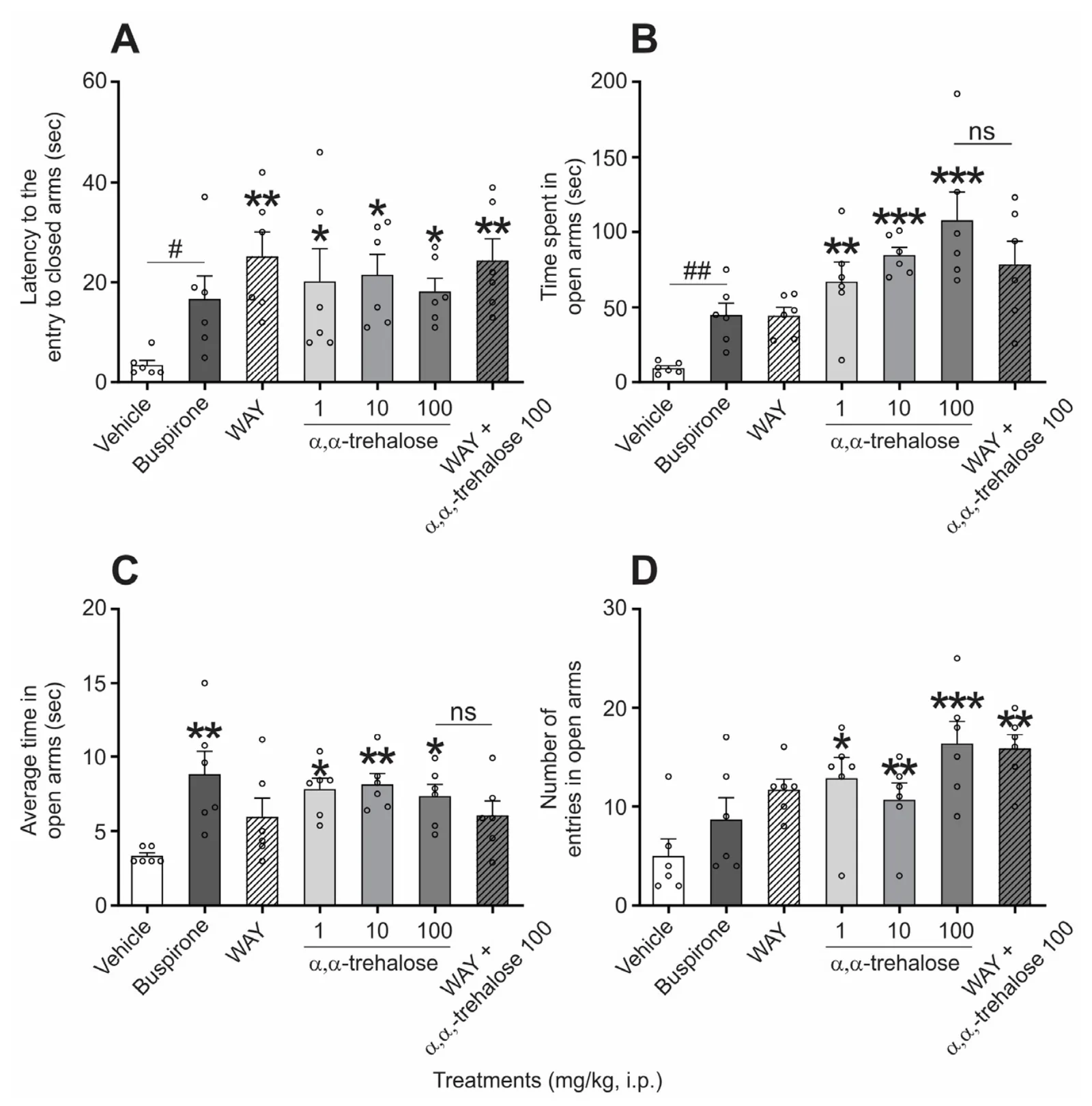
Anxiolytic-like effects of α,α-trehalose (1, 10 and 100 mg/kg, i.p.) and the reference drug buspirone (4 mg/kg, i.p.), as well as the presence of the antagonist of the 5-HT_1A_ serotonin receptor WAY-100635 (WAY, 1 mg/kg, i.p.) alone and combined with α,α-trehalose (100 mg/kg, i.p.) in the elevated plus-maze test to evaluate the following: (**A**) latency to the entry to closed arms, (**B**) time spent in open arms, (**C**) average time in open arms, and (**D**) number of entries in open arms. Bars represent the mean ± S.E.M., *n* = 6 per group. One-way ANOVA followed by Dunnett’s test; * *p* < 0.05, ** *p* < 0.01, and *** *p* < 0.001; ^#^ *p* < 0.05 and ^##^ *p* < 0.01. ns: no significant.

**Figure 5:**
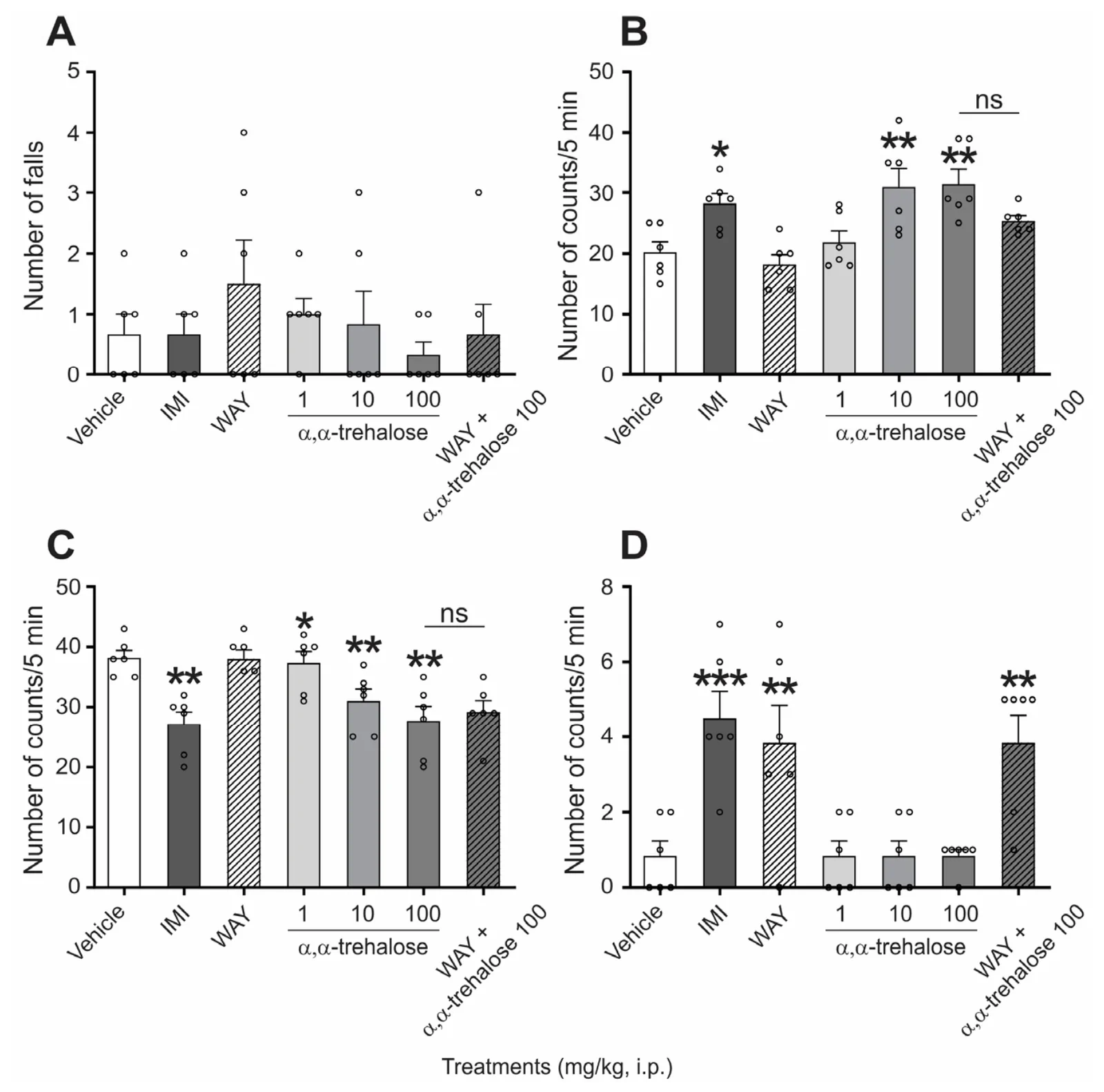
Antidepressant-like effects of α,α-trehalose (1, 10, 100 mg/kg, i.p.) and the reference antidepressant drug imipramine (IMI, 10 mg/kg, i.p.), as well as the presence of the antagonist of the 5-HT_1A_ serotonin receptor WAY-100635 (WAY, 1 mg/kg, i.p.) alone and combined with α,α-trehalose (100 mg/kg, i.p.) assessed in (**A**) the rota-rod test and the forced swim test to evaluate (**B**) immobility, (**C**) swimming, and (**D**) climbing behaviors in mice. Bars represent the mean ± S.E.M., *n* = 6 per group. One-way ANOVA followed by Dunnett’s test; * *p* < 0.05, ** *p* < 0.01 and *** *p* < 0.001. ns: no significant.

**Figure 6:**
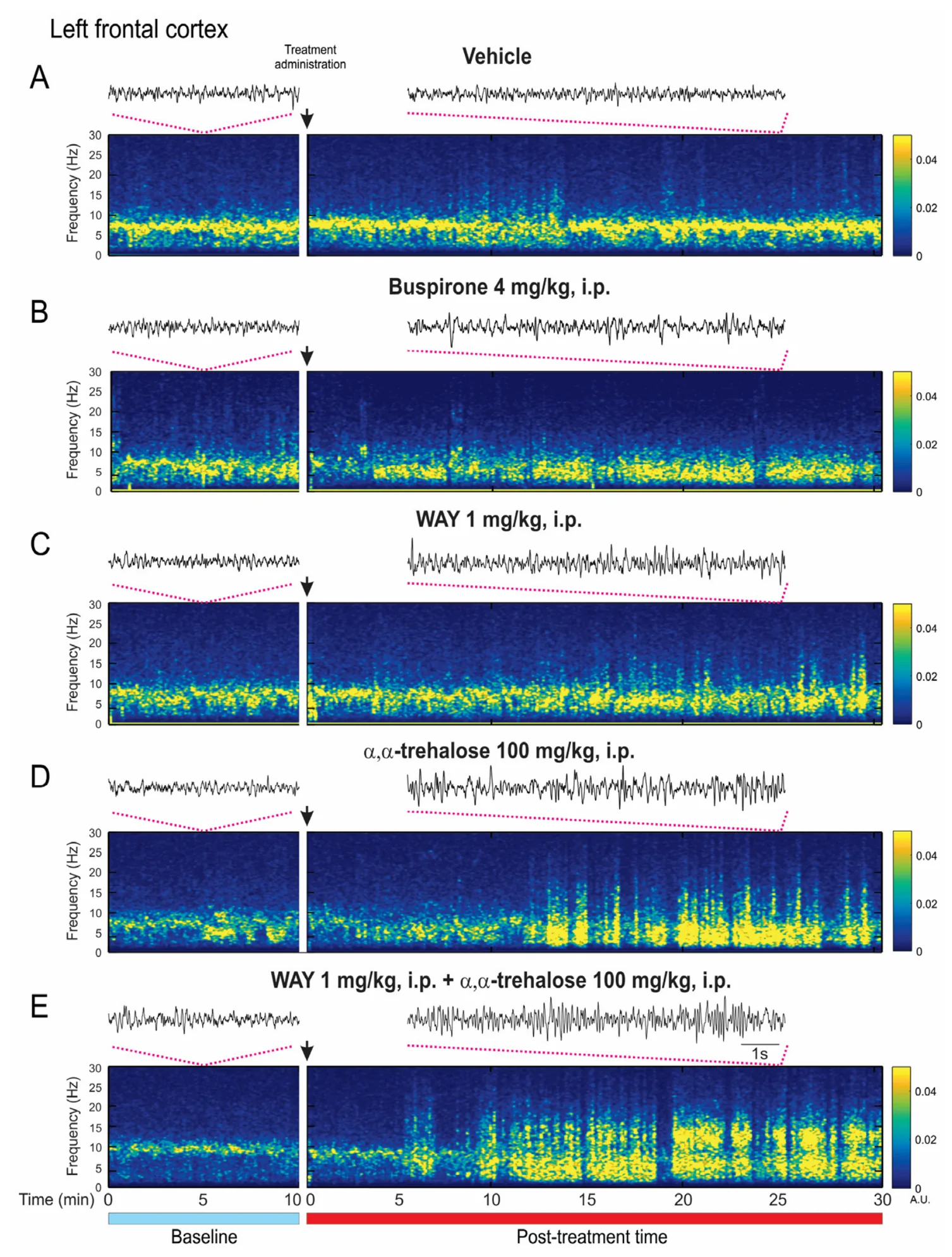
Representative multitaper spectrograms of ECoG activity recorded from the left frontal cortex of mice treated with (**A**) vehicle, (**B**) buspirone (4 mg/kg, i.p.), (**C**) WAY-100635 (WAY, 1 mg/kg, i.p.), (**D**) α,α-trehalose (100 mg/kg, i.p.), and (**E**) WAY (1 mg/kg, i.p.) + α,α-trehalose (100 mg/kg, i.p.). Representative ECoG traces are shown above each panel, corresponding to baseline and 25 min post-treatment, as indicated by black arrows.

**Figure 7:**
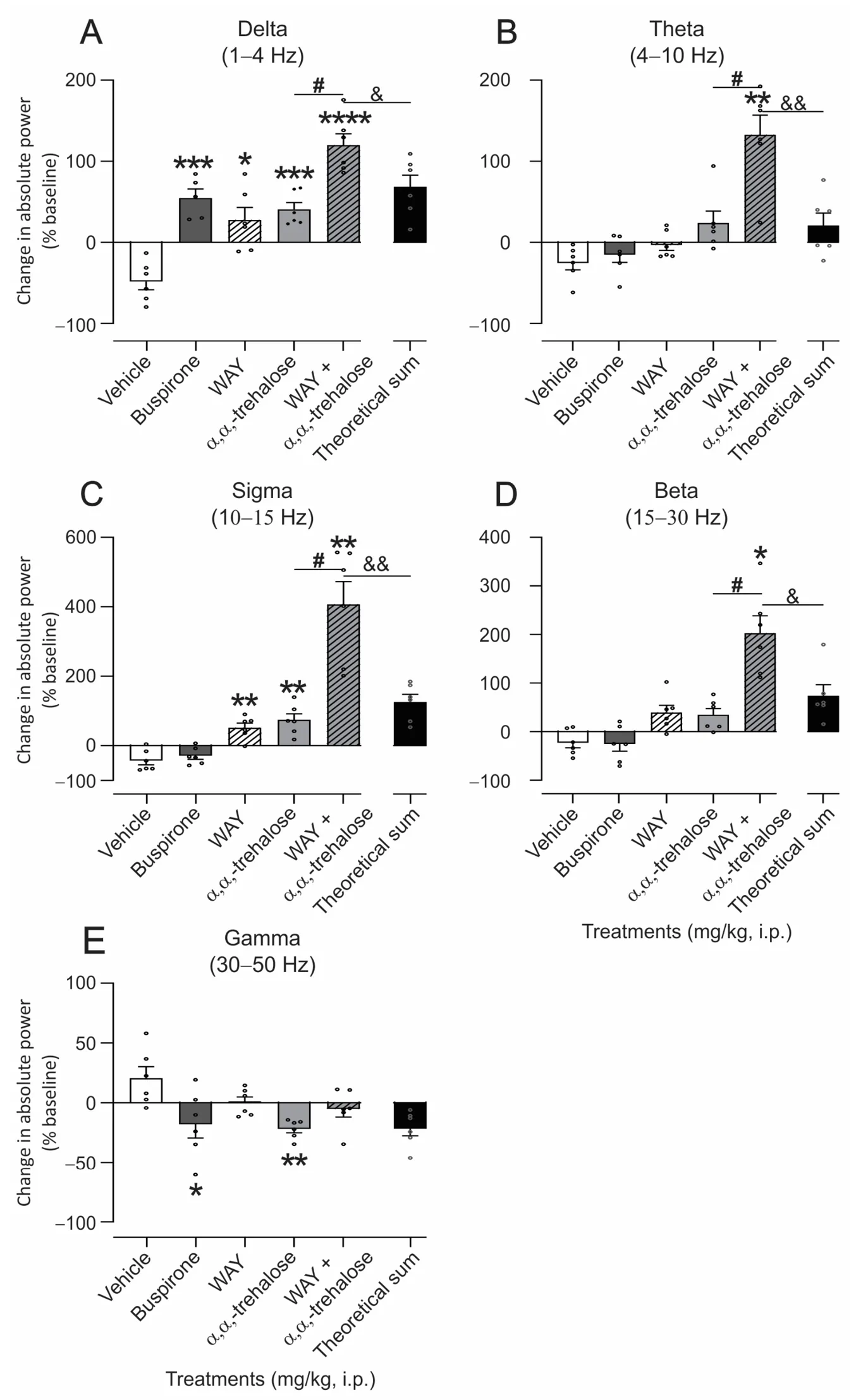
Normalized baseline-related changes in spectral power recorded from the left frontal cortex following the administration of vehicle, buspirone (4 mg/kg, i.p.), WAY-100635 (WAY, 1 mg/kg, i.p.), α,α-trehalose (100 mg/kg, i.p.) or the combination of WAY (1 mg/kg, i.p.) + α,α-trehalose (100 mg/kg, i.p.) in mice. Spectral power changes are shown for the (**A**) delta, (**B**) theta, (**C**) sigma, (**D**) beta, and (**E**) gamma (30–50 Hz) frequency bands at 30 min post-treatment relative to baseline. Data represent the mean ± S.E.M. (*n* = 6 animals per group). Statistical analysis was performed using one-way ANOVA followed by Tamhane’s T2 post hoc test; * *p* < 0.05, ** *p* < 0.01, *** *p* < 0.001, **** *p* < 0.0001 vs. vehicle; ^#^ *p* < 0.05 vs. WAY +α,α-trehalose group. The theoretical sum represents the expected additive response calculated from the individual effects of WAY and α,α-trehalose and was compared with the observed combined response using Student’s *t*-test; ^&^ *p* < 0.05 and ^&&^ *p* < 0.01 vs. WAY + α,α-trehalose.

**Figure 8:**
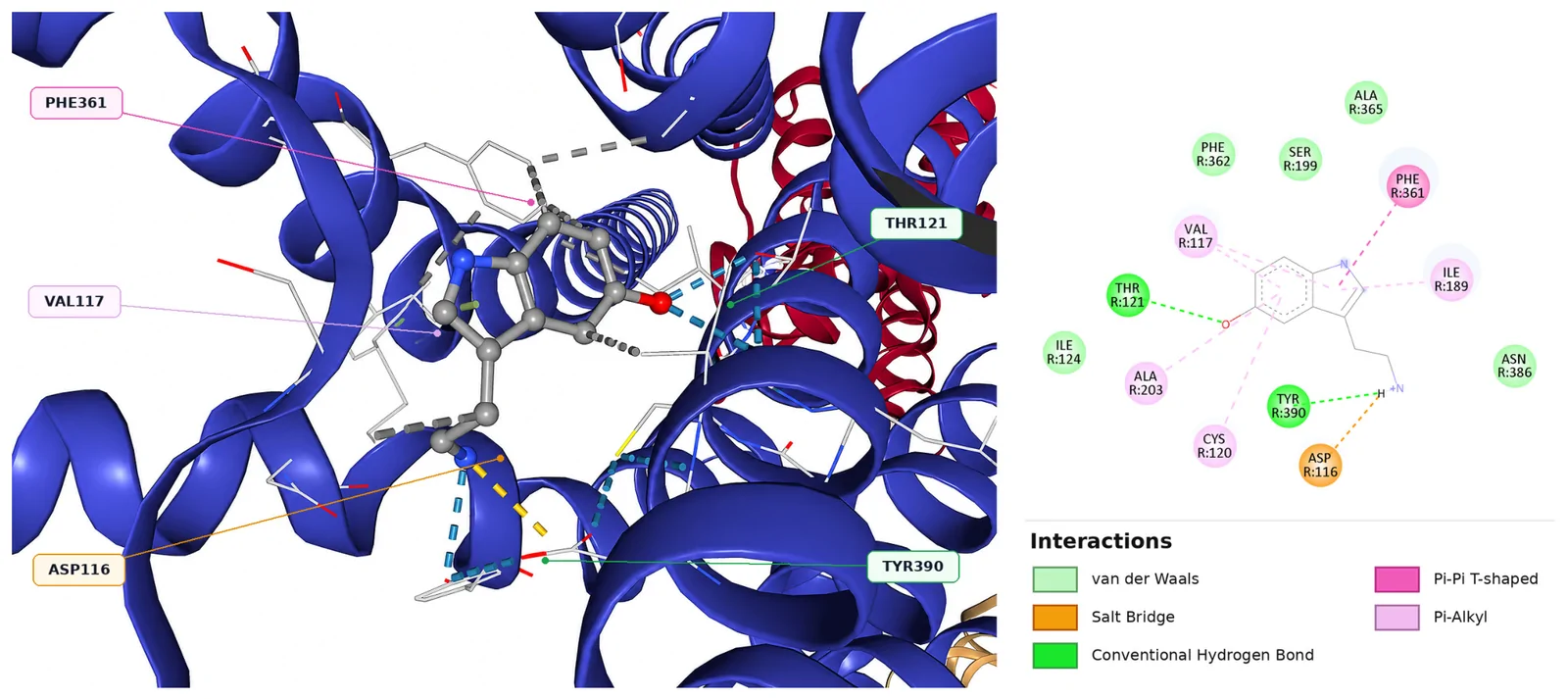
Predicted binding pose of serotonin (5-HT) within the orthosteric site of the 5-HT_1A_ receptor (PDB ID: 7E2Y) used as the orthosteric reference. Three-dimensional view of the top-ranked docking pose (left panel). Two-dimensional interaction diagram (right panel) showing conventional hydrogen bonds with Thr121 and Tyr390, a salt bridge with Asp116, a π–π T-shaped interaction with Phe361, and π–alkyl contacts (Vina affinity score = −6.1 kcal/mol).

**Figure 9:**
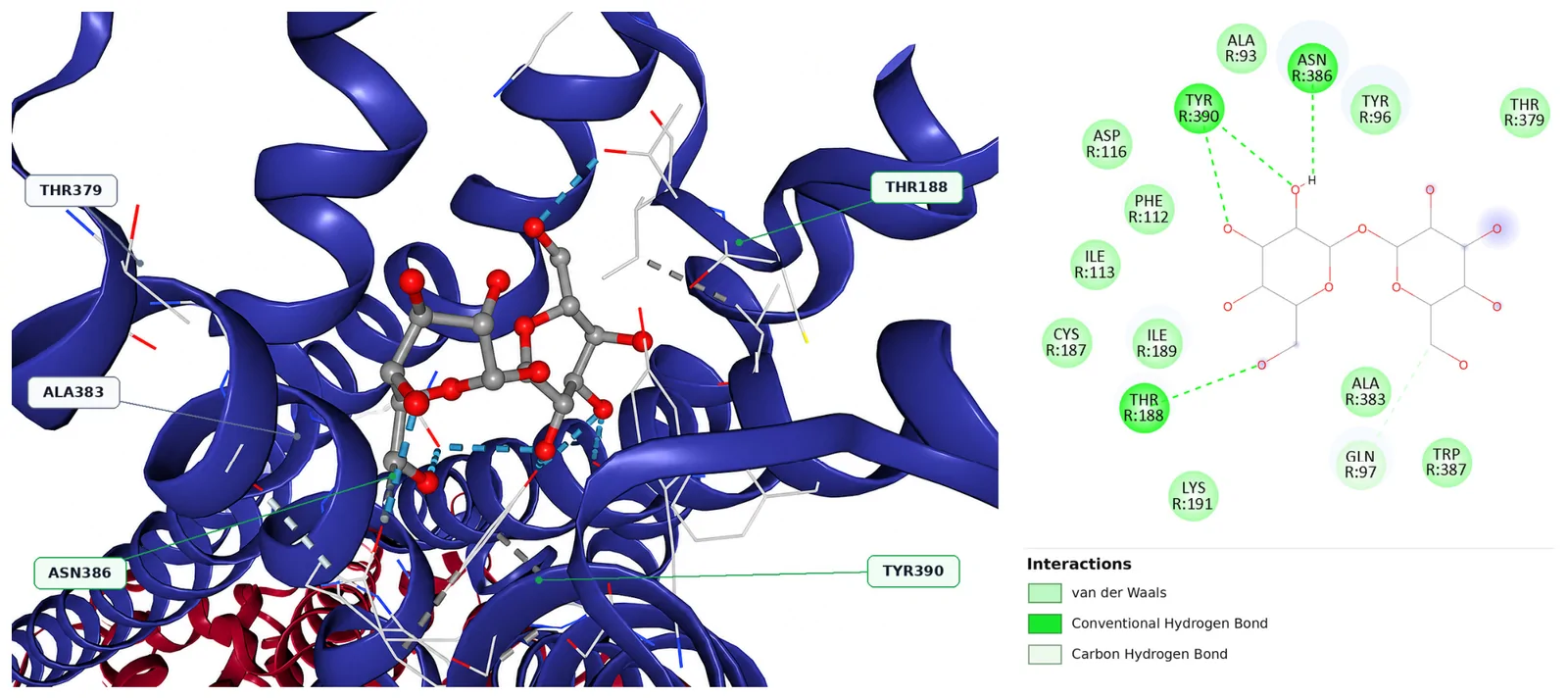
Predicted peripheral binding pose of α,α-trehalose at the 5-HT_1A_ serotonin receptor. Three-dimensional view (left panel) showing the outward position of α,α-trehalose near the extracellular entrance of the binding cavity. Two-dimensional interaction map (right panel) showing predicted conventional hydrogen bonds with Thr188, Asn386, and Tyr390, alongside surrounding non-covalent contacts including Thr379 and Ala383 (Vina affinity score = −6.3 kcal/mol). Note that this pose represents a peripheral and noncanonical binding mode and should not be interpreted as evidence of an experimentally validated allosteric site.

**Figure 10:**
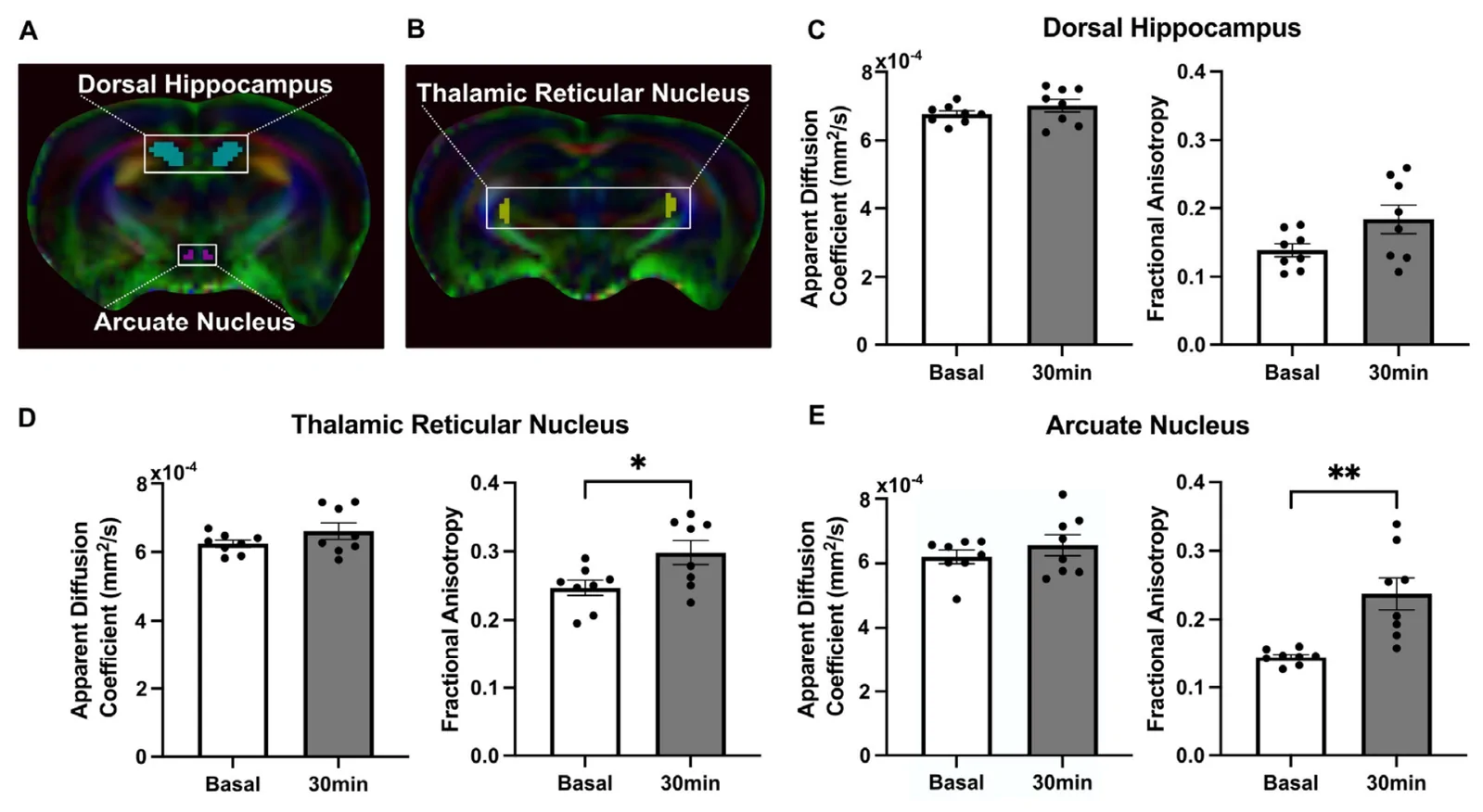
Fractional anisotropy (FA) and apparent diffusion coefficient (ADC) values from the Dorsal Hippocampus (DH), the Thalamic Reticular Nucleus (TRN), and the Arcuate Nucleus (AN). The values were obtained at two times: two days before the administration of α,α-trehalose (baseline exploration) and 30 min after a dose of 100 mg/kg, i.p. (**A**) Representative coronal image showing the evaluated brain regions (blue, DH; pink, AN) and (**B**) The TRN. The graphs show data obtained using in vivo diffusion tensor imaging. α,α-Trehalose treatment shows significantly increased FA values in (**D**) the TRN and (**E**) the AN. No changes were observed for ADC values in DH (**C**). Data are represented as the mean ± S.E.M. and analyzed by Student’s *t* test, * *p* < 0.05 and ** *p* < 0.01.

## Data Availability

Spectral data and other data supporting the findings are available from the corresponding authors upon reasonable request.

## Supplementary Materials

The following supporting information can be downloaded at: https://www.mdpi.com/article/10.3390/molecules31193383/s1, Figure S1: (A) α,α-trehalose from *Psilocybe cubensis* and (B) α,α-trehalose (Sigma-Aldrich), ^1^H (400 MHz), D_2_O; Figure S2: (A) α,α-trehalose from *Psilocybe cubensis* and (B) α,α-trehalose (Sigma-Aldrich), ^13^C (100 MHz), D_2_O; Figure S3: qHNMR for α,α-trehalose in DMSO-d_6_ with the addition of 1,4-dinitrobenzene (98 % pure) as Internal Calibrant. Refs. [75,76] are cited in the Supplementary Materials.

## Abbreviations

The following abbreviations are used in this manuscript:

Na_2_SO_4_	Anhydrous Sodium Sulfate
ADC	Apparent Diffusion Coefficient
AN	Arcuate Nucleus
ANOVA	Analysis of Variance
AQ	Aqueous Fraction
AP	Anteroposterior
CC	Chromatographic Column
CNS	Central Nervous System
DH	Dorsal Hippocampus
DTI	Diffusion Tensor Imaging
DWI	Diffusion-Weighted Imaging
ECoG	Electrocorticography
EEG	Electroencephalography
FA	Fractional Anisotropy
FFT	Fast Fourier Transform
FOV	Field of View
FST	Forced Swimming Test
GABA	Gamma-Aminobutyric Acid
GABA_A_	Gamma-Aminobutyric Acid Type A Receptor
Glc	Glucose
HPA	Hypothalamic–Pituitary–Adrenal
HPLC	High-Performance Liquid Chromatography
IMI	Imipramine
INPRFM	Instituto Nacional de Psiquiatría Ramón de la Fuente Muñiz
i.p.	Intraperitoneal
IR	Infrared Spectroscopy
KBr	Bromide Potassium
MDSs	Molecular Dynamics Simulations
MeOH	Methanol
ML	Mediolateral
MM-GBSA	Molecular Mechanics–Generalized Born Surface Area
m.p.	Melting Point
MRI	Magnetic Resonance Imaging
NMR	Nuclear Magnetic Resonance
OF	Organic Fraction
p.o.	Oral route
PDB	Protein Data Bank
ROS	Reactive Oxygen Species
S.E.M.	Standard Error of the Mean
5-HT	Serotonin
5-HT_1A_	Serotonin Subtype 1A Receptor
SSRIs	Selective Serotonin Reuptake Inhibitors
SNRIs	Serotonin and Norepinephrine Reuptake Inhibitors
TCAs	Tricyclic Antidepressants
TRN	Thalamic Reticular Nucleus
UAEH	Universidad Autónoma del Estado de Hidalgo
WAY	WAY-100635
WHO	World Health Organization
