# Supplementary material for: Preclinical Anxiolytic- and Antidepressant-like Effects of α,α-Trehalose, a Bioactive Compound Identified in Psilocybe cubensis
Source: Molecules. 2026 Sep 23;31(19):3383. doi: 10.3390/molecules31193383 (PMC13666409; doi:10.3390/molecules31193383)
Supplement: Supplementary file 1 [file molecules-31-03383-s001.zip › molecules-4555559-supplementary.pdf]

Supplementary material

Preclinical Anxiolytic- and Antidepressant-like Effects of α,α-Trehalose, a Bioactive Compound Identified in *Psilocybe cubensis*

Aylín Rocío Tabal-Robles[1,2], María Eva González-Trujano[1]*, Gabriel Fernando Moreno-Pérez[1], Alberto Hernandez-Leon[1], Susana Rojas-Lima[2], David Martínez-Vargas[3], Hiram Luna-Munguía[4], Raúl Iván Escamilla-Orozco[5], Laura Guzmán-Dávalos[6], José G. Alvarado-Rodríguez[2], J. Martín Torres-Valencia[2]*.

1 Laboratorio de Neurofarmacología de Productos Naturales, Dirección de Investigaciones Biomédicas en Salud Mental, Instituto Nacional de Psiquiatría Ramón de la Fuente Muñiz, Calz. México-Xochimilco 101, Col. San Lorenzo Huipulco, Tlalpan, Ciudad de México 14370, Mexico; ta260266@uaeh.edu.mx (A.R.T.-R.); mmspoodles@gmail.com (G.F.M.-P.); albertoh-leon@hotmail.com (A.H.-L.)

2 Área Académica de Química, Universidad Autónoma del Estado de Hidalgo, Km. 4.5 Carretera Pachuca-Tulancingo, Mineral de la Reforma 42184, Mexico; jgar@uaeh.edu.mx (J.G.A.-R.)

3 Laboratorio de Neurofisiología del Control y la Regulación, Dirección de Investigaciones Biomédicas en Salud Mental, Instituto Nacional de Psiquiatría Ramón de la Fuente Muñiz, Calz. México-Xochimilco 101, Col. San Lorenzo Huipulco, Tlalpan, Ciudad de México 14370, Mexico; davmv2@gmail.com

4 Departamento de Neurobiología Conductual y Cognitiva, Instituto de Neurobiología, Universidad Na-cional Autónoma de México, Campus UNAM-Juriquilla, Querétaro 76230, Mexico; hi-ram_luna@yahoo.com

5 Servicios Clínicos, Dirección de Servicios Clínicos, Instituto Nacional de Psiquiatría Ramón de la Fuente Muñiz, Calz. México-Xochimilco 101, Col. San Lorenzo Huipulco, Tlalpan, Ciudad de México 14370, Mexico; rescam01@gmail.com

6 Departamento de Botánica y Zoología, Universidad de Guadalajara, Zapopan 45200, Mexico; laura.guzman@academicos.udg.mx

*Correspondence: evagontru@yahoo.com.mx (M.E.G.-T.); jmartin@uaeh.edu.mx (J.M.T.-V.)

Characterization and quantification of α,α-trehalose in methanol (MeOH) extract of *P. cubensis* mushrooms.

^{1}H and ^{13}C NMR spectra were directly compared with those of a commercial authentic sample of α,α-trehalose (Sigma-Aldrich) α,α-trehalose (Figures S1 and S2, panels A & B).

Following previously reported procedures [1, 2], quantitative ^{1}D ^{1}H NMR analyses were performed using 1,4-dinitrobenzene (98% purity, Aldrich) as an internal standard. Thus, 3.8 mg of MeOH extract and 1.4 mg of 1,4-dinitrobenzene were carefully weighed and dissolved in 0.7 mL of DMSO-d6. The solution was filtered and introduced into 5 mm NMR tubes. The ^{1}D ^{1}H spectrum was acquired with the following parameters: zg pulse at 90°, temperature of 25 °C, TD of 96,152 points with zero filling at 256K, 4 dummy scans, relaxation time of 60 s, acquisition time of 4 s, spectral window of 20 ppm, transmitter offset of 6.17 ppm, and 64 scans. Post-processing included line broadening of 0.1 Hz, zero filling at 254K, manual phase adjustment, and baseline correction using a 5th-order polynomial. Quantification of α,α-trehalose was done by integrating H-1 signal at 4.87 ppm (doublet, J = 3.5 Hz)(Figure S3).

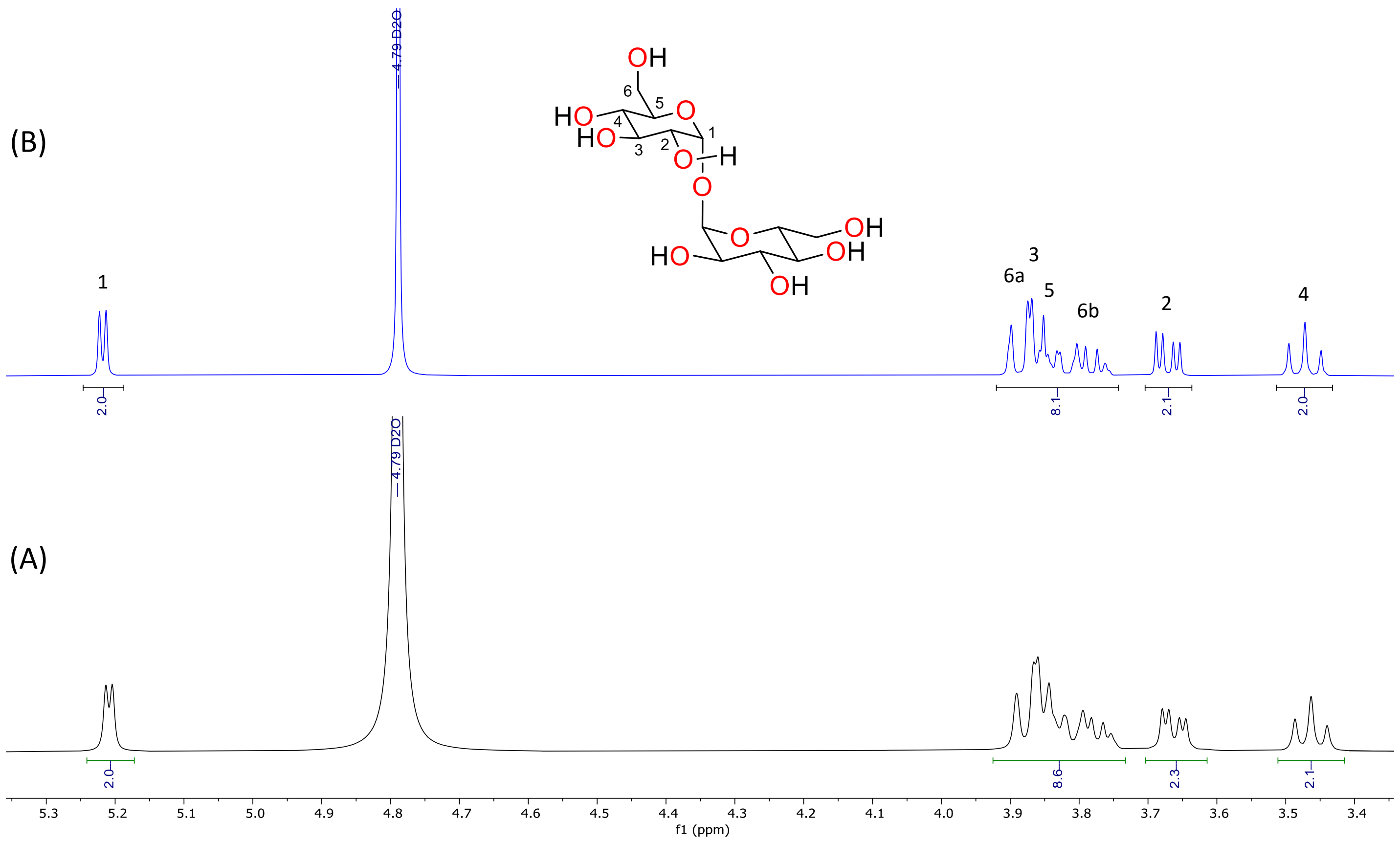

Figure S1. (A) α,α-trehalose from *Psilocybe cubensis* and (B) α,α-trehalose (Sigma-Aldrich), ^{1}H (400 MHz), D_2O.

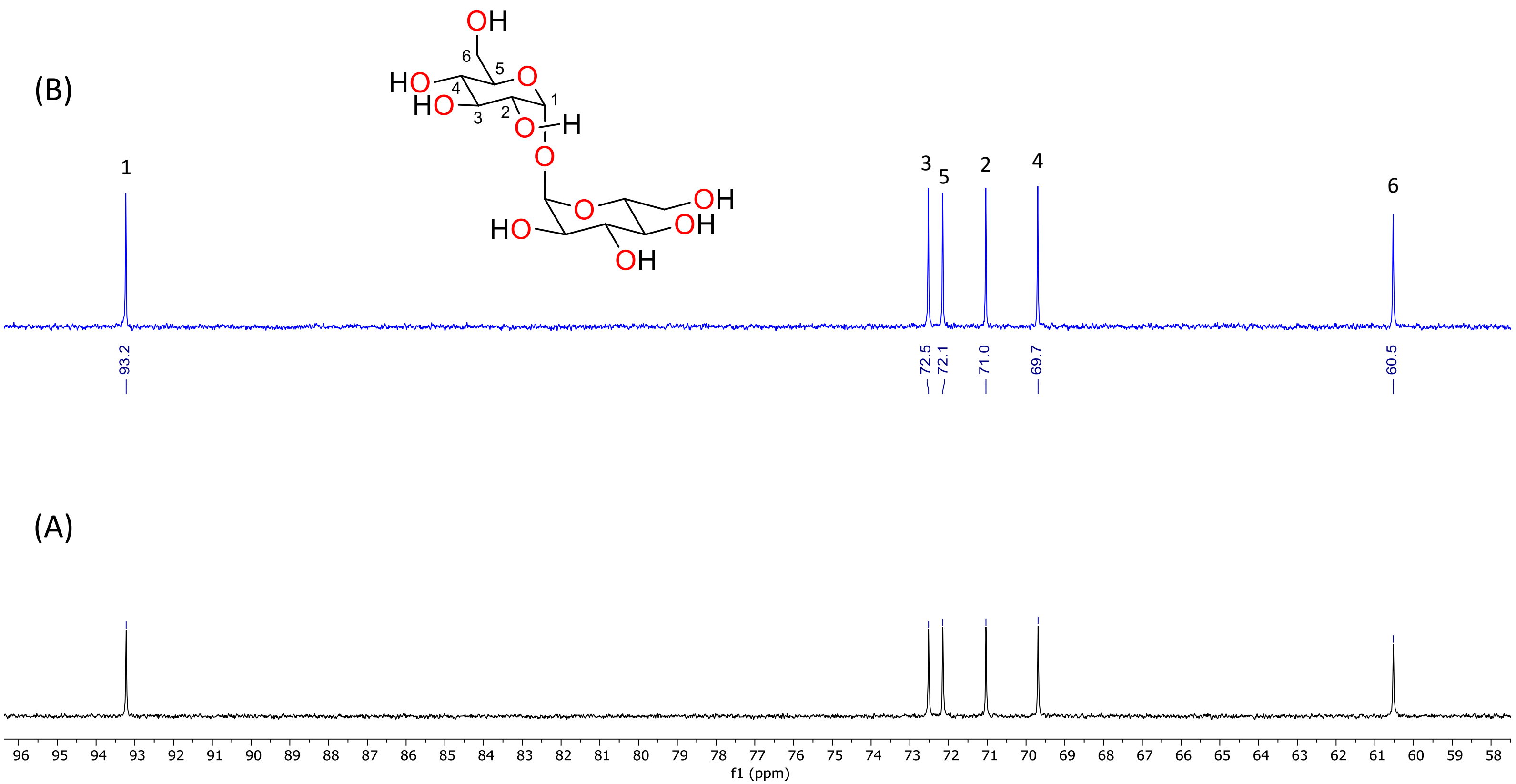

Figure S2. (A) α,α-trehalose from *Psilocybe cubensis* and (B) α,α-trehalose (Sigma-Aldrich), ^{13}C (100 MHz), D_2O.

**Step 1**	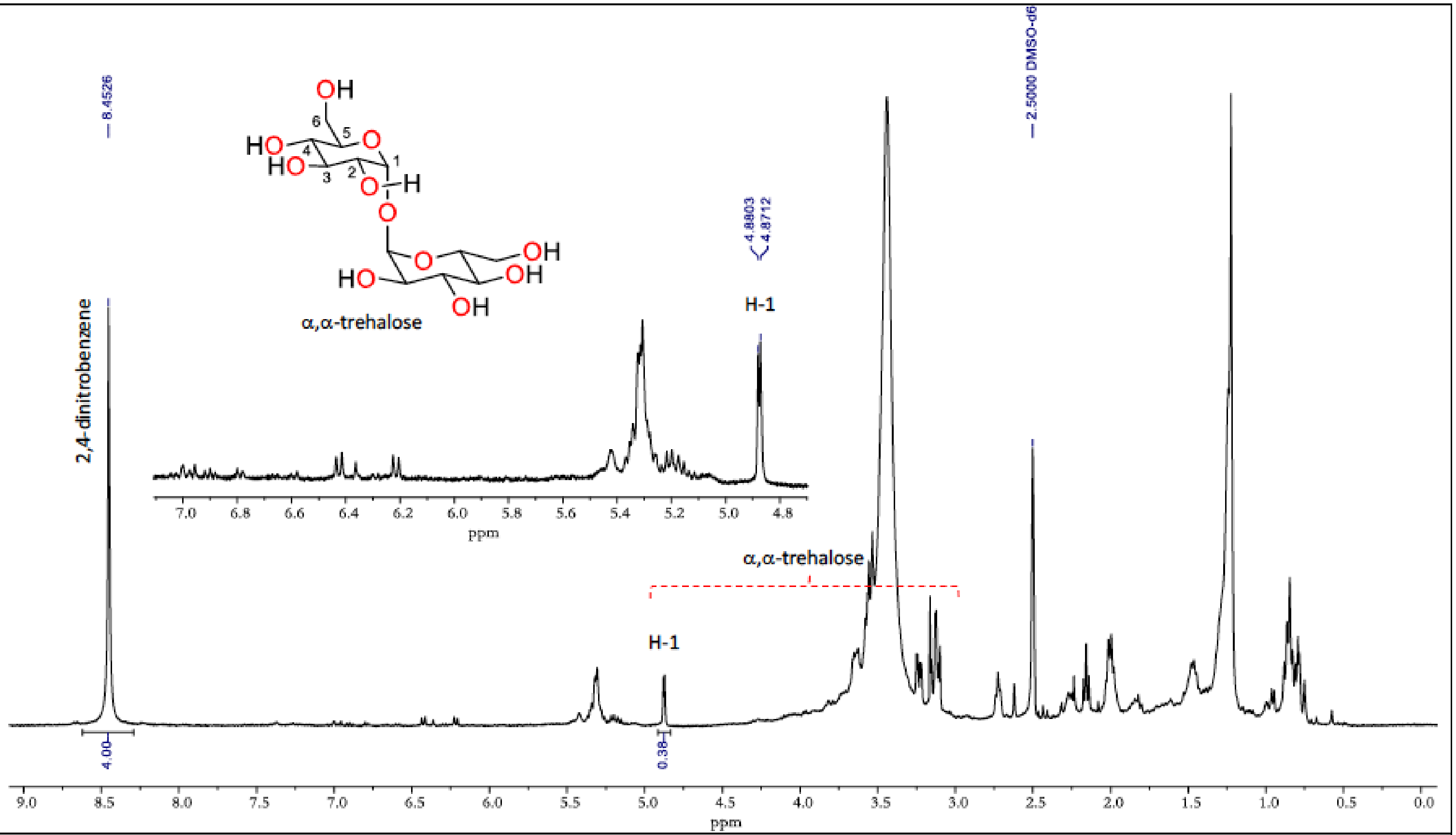
**Step 1**	$m_s = 3.8$ mg, $m_{IC} = 1.4$ mg, $P_{IC} = 98\%$
**Step 2**	$Int_t = 0.38$, $n_t = 2$
**Step 3**	$Int_{IC} = 4.00$, $n_{IC} = 4$
**Step 4**	$MW_t = 342.30$ g/mol, $MW_{IC} = 168.11$ g/mol
**Step 5**	$\alpha,\alpha\text{-trehalose } [\%] = \frac{n_{IC} \cdot Int_t \cdot MW_t \cdot m_{IC}}{n_t \cdot Int_{IC} \cdot MW_{IC} \cdot m_s} \cdot P_{IC} = 13.97\%$

Figure S3. qHNMR for α,α-trehalose in DMSO-d6 with the addition of 1,4-dinitrobenzene (98 % pure) as internal calibrant.

References

1. Cushman, M.; Georg, G.I.; Holzgrabe, U.; Wang, S. Absolute quantitative ^{1}H NMR spectroscopy for compound purity determination. *J. Med. Chem.* **2014**, *57*, 9219–9219, https://doi.org/10.1021/jm501683w.

2. Pauli, G.F.; Chen, S.-N.; Simmler, C.; Lankin, D.C.; Gödecke, T.; Jaki, B.U.; Friesen, J.B.; McAlpine, J.B.; Napolitano, J.G. Importance of purity evaluation and the potential of quantitative ^{1}H NMR as purity assay. *J. Med. Chem.* **2014**, *57*, 9220–923, https://doi.org/10.1021/jm500734a.
